# Achievements, challenges, and future prospects for industrialization of perovskite solar cells

**DOI:** 10.1038/s41377-024-01461-x

**Published:** 2024-09-03

**Authors:** Chuang Yang, Wenjing Hu, Jiale Liu, Chuanzhou Han, Qiaojiao Gao, Anyi Mei, Yinhua Zhou, Fengwan Guo, Hongwei Han

**Affiliations:** 1 Michael Grätzel Center for Mesoscopic Solar Cells, Wuhan National Laboratory for Optoelectronics, Key Laboratory of Materials Chemistry for Energy Conversion and Storage of Ministry of Education, Huazhong University of Science and Technology, Wuhan, 430074 Hubei China; 2 Collaborative Innovation Center for Advanced Organic Chemical Materials, Co-constructed by the Province and Ministry of Education Key Laboratory for the Synthesis and Application of Organic Functional Molecules, College of Chemistry and Chemical Engineering, Hubei University, Wuhan, 430062 Hubei China

**Keywords:** Solar energy and photovoltaic technology, Photonic devices

## Abstract

In just over a decade, certified single-junction perovskite solar cells (PSCs) boast an impressive power conversion efficiency (PCE) of 26.1%. Such outstanding performance makes it highly viable for further development. Here, we have meticulously outlined challenges that arose during the industrialization of PSCs and proposed their corresponding solutions based on extensive research. We discussed the main challenges in this field including technological limitations, multi-scenario applications, sustainable development, etc. Mature photovoltaic solutions provide the perovskite community with invaluable insights for overcoming the challenges of industrialization. In the upcoming stages of PSCs advancement, it has become evident that addressing the challenges concerning long-term stability and sustainability is paramount. In this manner, we can facilitate a more effective integration of PSCs into our daily lives.

## Abstract (web summary)

This review summarized the challenges in the industrialization of perovskite solar cells (PSCs), encompassing technological limitations, multi-scenario applications, and sustainable development. Additionally, the focus of future advancements in PSCs was discussed.

## Graphical abstract

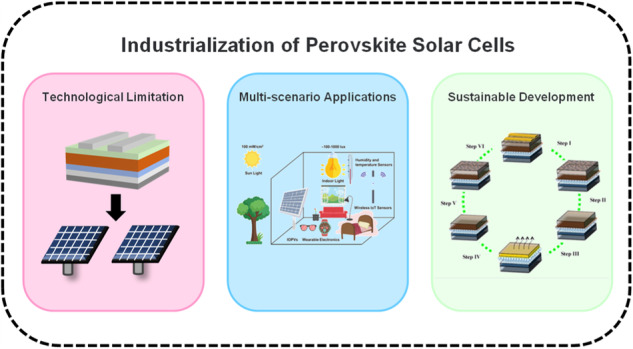

## Introduction

Solar power has consistently emerged as one of the most promising, reliable, and renewable energy sources among various alternatives^1,2^. Since the discovery of the photovoltaic (PV) effect, solar cell technology has continued to evolve and advance, enabling the widespread adoption of solar power as a viable renewable resource^3^. Currently, silicon solar cells occupy a dominant position in the solar cell industry^4^. As alternative solar technologies, such as thin-film solar cells or perovskite solar cells (PSCs), continue to evolve, silicon solar cells are increasingly encountering competitive pressures in the market. These cutting-edge technologies hold the promise of delivering significant cost advantages and enhanced performance, sparking intense ongoing research efforts.

**Fig. 1:**
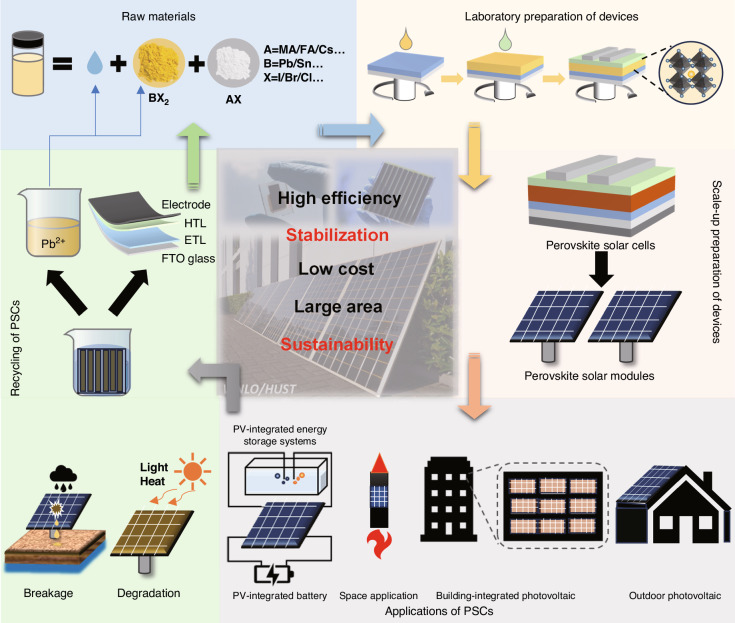
The life cycle of perovskite materials. Reproduced with permission from ref. ^140^ Copyright 2018, American Chemical Society

Metal halide perovskite materials have garnered significant interest as highly promising materials for photovoltaic devices due to their exceptional photoelectric properties^5,6^. These materials have captivated researchers and industry alike, as they offer great potential for advancing the field of photovoltaics. Following the initial fabrication of PSCs and their achievement with a power conversion efficiency (PCE) of 3.8%, research on PSCs has gained tremendous momentum^7^. With the persistent efforts of scientists, certified single-junction PSCs now boast a soul-stirring PCE of 26.1% (~0.0513 cm^2^), which ushered in the dawn of the industrial development of PSCs^8^. As shown in Fig. 1, in order to enhance the industrial feasibility of PSCs, it is imperative to undertake a thorough investigation of their complete life cycle. The intricate journey begins with sourcing raw materials, where the composition of perovskite plays a crucial role in adjusting the bandgap and enhancing stability. Subsequently, the meticulous creation of small-area PSCs hinges on achieving a high-quality perovskite film and establishing precise energy level alignment between the perovskite absorption layer and the charge transport layer. Advancing along this path toward the industrialization of PSCs entails the necessary transition to Perovskite Solar Modules (PSMs). During this phase, the primary objective is to minimize efficiency losses resulting from device amplification, where a variety of amplification techniques have been explored. Moving forward, researchers’ focus expands to exploring the versatile deployment of PSCs across various applications and scenarios. This represents our ultimate goal in harnessing the potential of these solar technologies. Last but certainly not least, we must extend our considerations to a critical aspect—sustainability. As PSCs fulfill their primary function, it becomes imperative to thoughtfully contemplate recycling mechanisms and embrace sustainable practices. This holistic understanding and management of the entire life cycle are pivotal in unlocking the full industrial potential inherent in PSCs, making it not just an energy solution but also an environmentally responsible technology.

However, compared to established PV technology and market demand, issues of PSCs about long-term stability, such as degradation and performance fluctuations, persist as challenges that should be overcome^9^. Moreover, the industrial application of PSCs still faces challenges related to scale, cost, and sustainable development in the life cycle^10–12^. These issues have emerged as significant constraints, demanding careful consideration and innovative solutions.

The collective objective revolves around the development of efficient, stable, cost-effective, large-scale, and sustainable PSCs. In this review, we delve into the primary challenges associated with the industrialization of PSCs, encompassing technological limitations, application constraints, and sustainable development. For the technological limitations, although only a small difference exists between PSCs and silicon solar cells in terms of device efficiency, there is a big gap in long-term stability. In addition, reducing the efficiency sacrifice brought by the device amplification process is also urgent to be addressed. Within the realm of application constraints, the crucial step toward enabling the diverse applications of PSCs across various scenarios lies in extending the device’s lifespan and optimizing its production cost so that it can maximize economic advantages. Ultimately, the reduction of pollution generated during PSC preparation remains paramount for fostering sustainable development.

## Technology limitations

PSCs have gained prominence as the focus of research in the solar energy sector. Nevertheless, numerous challenges still persist, encompassing the need for continued efficiency enhancement, bolstered stability, and the establishment of scalable PSC production methods. Within this chapter, we will comprehensively address these concerns, expounding on each one and providing an overview of current remedial approaches.

### The PCE and improvement strategies

The general structural formula of 3D organic-inorganic hybrid perovskite is ABX_3_, where A-site ion in perovskite compounds comprises methylamine ion (MA^+^), formamidine ion (FA^+^), or alkali metal ion, while the B-site ion consists of Pb^2+^ or Sn^2+^, and X-site ions represent halogen ions. The structural diversity and huge composition space of perovskite make it possible to achieve a series of functional properties. Up to now, the highest PCE of single-junction PSCs reached 26.1%, which is comparable to that of monocrystalline silicon solar cells. However, opportunities for further improvement remain on the path towards achieving the Shockley-Queisser (S-Q) limit^13^. Based on the existing research, fine-tuning the optical bandgap of perovskite materials through composition engineering involving multicomponent A- and X-site ions offers a direct means of customizing the inherent characteristics of perovskite. This approach holds the potential to yield efficient and high-performance PSCs^14^. Besides, augmenting the PCE primarily encompasses the enhancement of crystal quality, the passivation of defects, the facilitation of charge extraction at interfaces, and other related measures.

#### The enhancement of crystal quality

Elevating the crystalline quality of perovskite films holds significant potential for enhancing the overall performance of PSCs. The LaMer model provides valuable insights into the nucleation and grain growth process of crystal participated from precursor solution^15,16^. Based on this theory, when the solvent’s evaporation rate is slow, the solution concentration gradually approaches the critical level over an extended period. This results in a low concentration of nuclei, allowing initial nuclei ample time and space to grow into larger crystals. While this fosters perovskite films with minimal grain boundaries, film coverage may be inadequate during this phase. Conversely, a high solvent evaporation rate swiftly elevates the solution concentration above the critical level. In this scenario, the concentration of atomic nuclei is high, limiting the time and space for their growth. This high concentration facilitates the formation of a uniform and fine perovskite film, albeit with an increased presence of grain boundaries^17^. Therefore, precise control of the solvent evaporation rate is crucial for achieving perovskite films with optimal uniformity, coverage, and roughness.

From the reported literature we can see that the formation of immediate phase can effectively decelerate the crystallization process, facilitating the growth of crystals with large size. The induction of the immediate phase can be realized either through solvents with high coordination ability or through the incorporation of additives^18–20^.

**Fig. 2:**
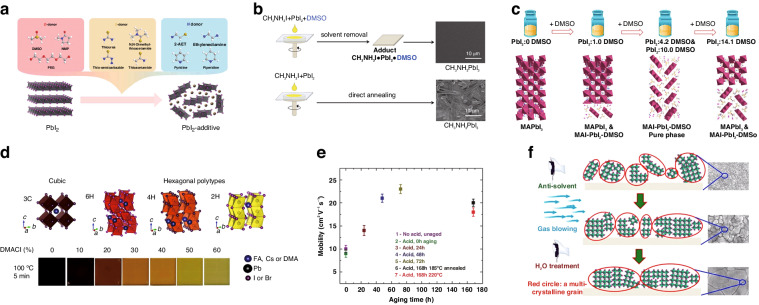
The methods for enhancing the quality of perovskite film. **a** Schematic diagram of Lewis acid-base interaction between solvent or additive and PbI_2_. Reproduced with permission from ref. ^21^ Copyright 2019 Wiley-VCH. **b** Perovskite films prepared by one-step method and their SEM images. Reproduced with permission from ref. ^22^ Copyright 2016 American Chemical Society. **c** Schematic diagram of the relationship between the content of DMSO and the composition of the intermediate phase. Reproduced with permission from ref. ^23^ Copyright 2017 Elsevier Ltd. **d** Photographs of DMA_x_(FA_0.83_Cs_0.17_)_1–x_Pb(Br_0.2_I_0.8_)_3_Cl_x_ perovskite films treated by different amounts of DMACl and their corresponding crystal structure. Reproduced with permission from ref. ^24^ Copyright 2022 Springer Nature. **e** The effective charge-carrier mobilities of perovskite films prepared by precursor solution with different concentrations of colloids. Reproduced with permission from ref. ^26^ Copyright 2017 Wiley-VCH. **f** Crystal structure simulation and SEM images under different processing methods. Reproduced with permission from ref. ^30^ Copyright 2018 American Chemical Society

Lead iodide (PbI_2_) is the precursor material of perovskite, which could be functioned as a Lewis acid. Solvents such as dimethyl sulfoxide (DMSO), thiourea, and pyridine furnish lone pairs of electrons, which can be classified as Lewis base. The interaction between these solvents and PbI_2_ can give rise to Lewis acid-base interaction, as shown in Fig. 2a^21^. For instance, N,N-dimethylformamide (DMF) serves as a frequently used solvent in perovskite precursor solutions, which was applied to examine the effect of DMSO on the growth of perovskite films. Although the DMF molecular can be coordinated with PbI_2_, perovskite films produced through the conventional one-step method displayed needle-like morphology, leaving the substrate incompletely covered. This can be attributed to the comparatively lower coordination ability of DMF in comparison to DMSO, which impeded DMF from effectively postponing the MAI-PbI_2_ reaction. The introduction of equimolar DMSO to DMF can lead to the formation of an intermediate phase, MAI-PbI_2_-DMSO, which effectively mitigates the rapid self-assembly crystallization stemming from the direct MAI-PbI_2_ reaction. Following the volatilization of DMSO, a highly uniform MAPbI_3_ film is meticulously generated (Fig. 2b)^22^. The inclusion of DMSO can indeed facilitate immediate phase formation and prolong the crystallization process. However, it should be known that an increase in the content of DMSO may not necessarily lead to improved outcomes. Through strategic adjustments in the PbI_2_/DMSO ratio, a sequence of transformative phases within the immediate phase film can be observed as the content of DMSO increases. The transition occurred from a state of pure perovskite phase to a composite blend of perovskite/MA_2_Pb_3_I_8_(DMSO)_2_, progressing further to the distinct phase of pure MA_2_Pb_3_I_8_(DMSO)_2_, and ultimately to the combined MA_2_Pb_3_I_8_(DMSO)_2_/perovskite configuration (Fig. 2c). Notably, these diverse intermediate phases exhibited varying relative perovskite crystal structures and qualities. The intermediate phase of pure MA_2_Pb_3_I_8_(DMSO)_2_ exhibited a marked tendency for growth along the (110) direction. This growth behavior prompted the formation of perovskite film with reduced horizontal grain boundaries and decreased density of trap states. Consequently, this unique morphology translated into the highest PCE when evaluated within parallel group experiments. However, excessively slow crystallization rates resulting from high concentrations of DMSO led to irregular perovskite grain sizes and heightened surface roughness in the perovskite film^23^. Hence, solvent coordination ability should reside within an appropriate range so that it can achieve the optimal perovskite film with high crystallinity. Furthermore, the crystallization process can be influenced by factors such as solvent polarity, vapor pressure, boiling point, steric hindrance, and viscosity. Consequently, when choosing a solvent to regulate crystallization, it is essential to carefully consider how various solvent properties impact the crystallization process.

Beyond solvents, the incorporation of additives can also foster the development of the immediate phase. Dimethylammonium was utilized to control the intermediate phases in the perovskite precursor through a high-temperature processing technique, in the absence of DMSO (Fig. 2d). This method enabled precise control over the crystallization sequence, bringing about finely tuning in grain size, orientation, and overall crystallinity of the perovskite film, which resulted in fewer structural defects and higher PCE^24^.

The colloidal characteristics of the perovskite precursor solutions were observed to exhibit a direct correlation with both the defect concentration and crystallinity of the resulting perovskite film^25^. Hence, the interplay between an acidic additive and the dissolution of the colloidal framework has been established. Via facilitating the gradual dissolution of these colloids covering defined time regions, the nucleation, growth dynamics, and eventual morphology of perovskite film can be significantly modified. This enhancement in material quality fosters the reduction of microstrain and a remarkable increase in charge-carrier mobilities (Fig. 2e). Employing precursor solution with a meticulously optimal colloidal concentration can yield outstanding optoelectronic performance of PSCs^26^.

Additionally, some other methods are also applied to gain highly crystallized perovskite film, for instance, anti-solvent engineering (ASE), gas-assisted preparation, gas-blowing fabrication, or some other methods^27–29^. As shown in Fig. 2f, by synergizing the one-step ASE approach with subsequent gas blowing, the MAPbI_3_ film with high orientation and polycrystalline nanograins (150∼500 nm) can be effectively fabricated in the beginning. After being treated with anti-solvent-containing H_2_O, the perovskite grains were expanded to 1.5 μm. As a result, the PSCs reached an excellent PCE of 21% with a prominent fill factor (FF) of 86%^30^.

#### The passivation of defects

The reduced efficiencies compared to the theoretical counterparts can be attributed to discrepancies between the actual measured open circuit voltage (*V*_OC_) and FF. The decrement in *V*_OC_ and FF is linked to losses stemming from Shockley-Read-Hall (SRH) recombination, a consequence of volume and interface defects^31^. Hence, the passivation of defects holds paramount significance in minimizing recombination and enhancing the photovoltaic performance of devices.

Perovskite crystals own low defect-formation energy in thermodynamics^32^. For example, perovskite crystals harbor a substantial quantity of dangling bonds on surfaces, notably uncoordinated ions like Pb^2+^ or X^−^. These ions have the potential to act as defects in perovskite film^33^. The majority of defects reside within the shallow energy levels near the band edges, exhibiting electrical activity^34^. Conversely, anti-site and interstitial defects are positioned at deeper electronic levels, functioning as nonradiative recombination centers detrimental to device efficiency^35^.

Among the array of available methods, additive engineering has emerged as a remarkably efficient strategy for defect passivation. Generally speaking, the most potent approach for defect passivation involves leveraging Lewis acid-base interactions to passivate uncoordinated ions. The integration of Lewis acid and base additives into perovskite precursors can effectively reduce non-radiative recombination centers and defects at grain boundaries^36–38^.

##### Lewis acid

Lewis acid encompasses specific ions or even molecules that can function as electron pair acceptors, which can effectively mitigate undercoordinated I^−^ ions and Pb-I anti-site defects. Frequently employed Lewis acid additive mainly consists of cationic additives, fluorine-containing aromatic molecules, as well as fullerene and its derivatives^39–41^.

**Fig. 3:**
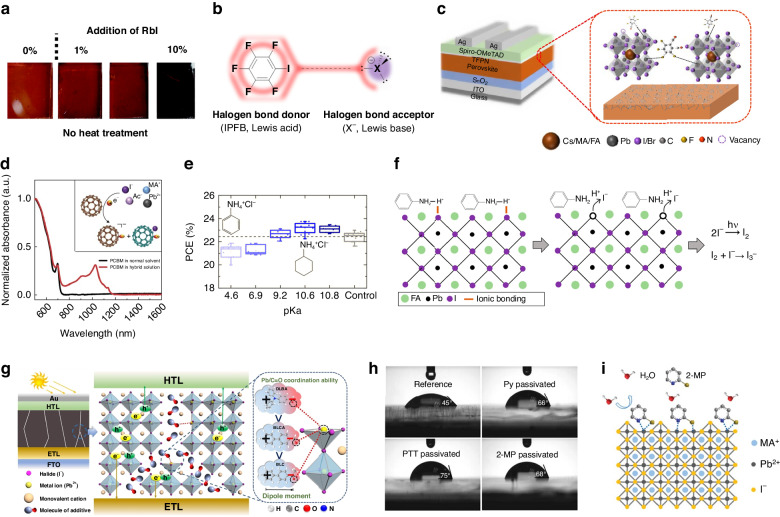
The defect passivation with Lewis acid and Lewis base. **a** Photographs of perovskite film with different addition of RbI (0, 1, 5, 10%) at room temperature. Reproduced with permission from ref. ^45^ Copyright Royal Society of Chemistry. **b** Schematic view of the halogen bond interaction between the IPFB and a generic halogen anion. Reproduced with permission from ref. ^41^ Copyright 2014 American Chemical Society. **c** Molecular structure of TFPN and its passivation diagram. Reproduced with permission from ref. ^47^ Copyright 2021 American Chemical Society. **d** UV absorption spectra of the hybrid solution show the interaction between PCBM and perovksite ions. The inset image shows the interaction between I^−^ and PCBM and the formation of PCBM radical anion and PCBM–halide radical. Reproduced with permission from ref. ^49^ Copyright 2015 Springer Nature. **e** The pKa value and PCE for different additives. **f** Schematic illustration of chemical reaction at perovskite surface with ANCl. Reproduced with permission from ref. ^54^ Copyright 2021 American Chemical Society. **g** Action mechanism diagram of additive dipole effect by DLBA, BLCA, and BLC on perovskite film. Reproduced with permission from ref. ^57^ Copyright 2023 Wiley-VCH. **h** Photographs of the unpassivated and passivated perovskite films before and after high humidity aging. **i** Passivation mechanism of perovskite treated by 2-MP. Reproduced with permission from ref. ^58^ Copyright 2019 Wiley-VCH

As for the cationic additive, given the valence distribution within the perovskite lattice and the redox stability of alkali metals, alkali metal cations with a positive charge are deemed optimal candidates for doping^42^. The incorporation of alkali metal cations (K^+^ and Na^+^) was proved to be an effective additive, leading to a significant enhancement in perovskite film quality with reduced grain boundaries and fewer trap states. This improvement resulted in an elevated built-in potential, ultimately resulting in higher PCE^43^. Besides, the incorporation of Rb^+^ dopants has been substantiated as an effective strategy for diminishing nonradiative recombination via chemical passivation and eradicating hysteresis in PSCs^44^. The δ-FAPbI_3_ can be suppressed by incorporating a mere 1% RbI into the precursor solution, employed for fabricating (FAPbI_3_)_0.83_(MAPbBr_3_)_0.17_ perovskite film (Fig. 3a). Impressively, samples containing Rb^+^ exhibited prolonged charge carrier lifetimes exceeding 1 μs, along with heightened *V*_OC_ and minimal current–voltage (J–V) hysteresis^45^.

Lewis acid of the fluorine-containing aromatic variety exhibits potent electronegativity due to the presence of fluorine atoms. These atoms adeptly induce the withdrawal of electron density from both the adjacent aromatic ring and the distal end, resulting in the development of a positive charge on this particular side (Fig. 3b). This unique interaction can effectively passivate defects through a non-covalent bonding effect^38,46^. For example, iodopentafluorobenzene (IPFB), wherein five fluorine atoms are strategically affixed to the five vertices of the benzene ring, was incorporated to coat the perovskite crystals. Fluorine engendered a reduction in electron density around the connected iodine atom within IPFB. This phenomenon led to a partial positive charge on the iodine, enabling it to establish a halogen bond with adjacent halogen atoms. Through this mechanism, uncoordinated halogen anti-site defects were effectively passivated^41^. Similarly, the tetrafluorophthalonitrile (TFPN) with four fluorine atoms was designed, which can interact with uncoordinated Pb^2+^, resulting in a proficient reduction of defect state density on the perovskite surface (Fig. 3c). Besides, cyanogroup within TFPN effectively ameliorates defects from uncoordinated Pb^2+^. Moreover, with the incorporation of TFPN at the interface, the Fermi level of the perovskite absorbent layer experienced a discernible shift of approximately 0.15 eV towards its valence band, prompting the creation of a positive dipole oriented towards the perovskite. Consequently, an amplified electric field effect ensued at the interface, considerably heightening the efficiency of hole extraction and transportation^47^.

Fullerene derivatives can also passivate defects, inhibit non-recombination, and improve film quality^48^. For example, Xu et al. found that the PCBM can passivate the iodide-rich trap sites on the surface when incorporated at or near perovskite grain boundaries, which can reduce hysteresis and promote electron extraction^49^ (Fig. 3d).

##### Lewis base

Lewis base refers to ions or molecules that act as electron pair donors, rendering it adept at passivating electron-deficient defects, such as uncoordinated Pb^2+^. The prevalent passivation groups utilized as Lewis base primarily encompass those functional groups comprising N-, O-, or S-atoms^50–52^.

N-donor Lewis base molecules effectively mitigate the presence of ionic charged defects, such as Pb^2+^ and I^−^, situated at grain boundaries or interfaces. This passivation is predominantly achieved through hydrogen bonding. In addition, amino-containing molecules with a certain steric hindrance can also anchor with perovskite to form low-dimensional perovskite, which can enhance the humidity stability of perovskite film. Tao et al. employed the new Lewis base additive, ethylenediamine chlorides (EDACl_2_). This compound proved instrumental in facilitating the generation of perovskite films with fewer trap states. Relative photoelectric assessments unveiled that the inclusion of EDACl_2_ enhanced the charge transport in the perovskite film, concurrently diminishing non-radiative recombination processes^53^. While N-donor molecules could effectively passivate uncoordinated Pb^2+^ ions in perovskite, it is noteworthy that the protonic characteristics of the passivating agent may also exert adverse effects on perovskite film. Park et al. presented a comprehensive analysis of the impact of acid dissociation constants (K_a_) in passivation agents on the photovoltaic performance of PSCs. A notable enhancement in PCE is observed when post-treated by cyclohexylammonium chloride (CYCl), the pKa value of which is 10.6. Conversely, the PCE experienced a decrease when treated with anilinium chloride (ANCl) with a relatively lower pKa value (4.6), primarily due to the unfavorable generation of increased traps induced by ANCl (Fig. 3e). This discrepancy in PCE was ascribed to the degree of deprotonation (pK_a_), which took a pivotal part in the formation of defect-mediated traps. The deprotonation process associated with lower-pK_a_ ANCl led to the release of free iodide (Fig. 3f), subsequently contributing to the emergence of iodide defects^54^. What’s more, the deprotonating effect of a highly basic Lewis base can lead to the deprotonation of the MA^+^ cations in MAPbI_3_ perovskite. This deprotonation process could potentially trigger the release of volatile organic molecules (FA and MA), consequently inducing lattice distortion or, in more severe cases, complete collapse of their crystal structures. The structural alteration ultimately contributed to the deterioration of the device’s performance^55^. Hence, when crafting N-donor Lewis base molecules, a judicious design approach must take account of the substantial implications of passivation and proton behavior to PSC devices.

Lewis base molecules with O-donor groups, such as carboxyl, exhibit a passivation effect akin to that of N-donor molecules. Iyer et al. introduced three additives, namely benzene carboxylic acid (BCA), benzene-1,3-dicarboxylic acid (BDCA), and benzene-1,3,5-tricarboxylic acid (BTCA), into the precursor solution. The incorporation of carboxylic acid moieties proved valid in modulating perovskite films, resulting in fewer trap states as well as ion migration. The presence of these additives during perovskite film formation exerted a profound effect on charge transfer dynamics, leading to enhanced performance and stability of the PSCs. Notably, devices incorporating BTCA exhibited the most remarkable outcomes, achieving the *V*_OC_ up to 1.076 V, marking an increase of about 80 mV^56^. While carbonyl molecules have demonstrated their efficacy as additives for facilitating the preparation of PSCs with high performance, the relationship between the structure and property of carbonyl agents and their capacity to passivate defects in perovskite films remains unclear. Hence, Pang et al. selected a variety of carbonyl additives featuring a single carbonyl group and a robust π-conjugate structure, which included Biphenyl-4-carboxaldehyde (BLCA), 4-Acetyl-biphenyl (BLC), and 4-(N, N-Diphenylamino)-benzaldehyde (DLBA). This selection aimed to investigate the intricate interaction between the functional groups of these additives and perovskite film. Their investigation revealed a positive correlation between the molecular dipole of the additives and their interaction with uncoordinated Pb^2+^ defects (Fig. 3g). A more pronounced molecular dipole was conducive to an enhanced passivation effect of the carbonyl additives. Among these organic molecules, DLBA exhibited the highest polarity, underscoring exceptional proficiency in passivating defects in perovskite film^57^. Therefore, it is necessary to take into account the effect on molecular polarity (charge density of functional groups) in perovskite films when designing O-donor molecules. However, whether higher polarity can achieve a better passivation effect and if there is a critical value still needs to be further investigated.

S-donor molecules act in a similar way to N- or O-donor molecules. Zhu et al. introduced a bidentate molecule, 2-mercaptopyridine (2-MP), to enhance anchoring ability, thereby simultaneously diminishing defects and elevating stability. In comparison to monodentate molecules like pyridine (PY) and p-toluenethiol (PTT), MAPbI_3_ film passivated by 2-MP exhibited a remarkable increase in photoluminescence (PL) lifetime and excellent thermal stability. What’s more, the unpassivated MAPbI_3_ experienced a rapid transition from a black film to a transparent state within a 30-min timeframe, while the humid stability exhibited a marginal enhancement for PY and PTT-passivated MAPbI_3_, where a few persistent black dots were observed after 1 h. Notably, MAPbI_3_ films passivated with 2-MP displayed unexpected resistance in a highly humid environment, showcasing minimal color alteration even after enduring concentrated moisture invasion for 5 h (Fig. 3h). This may be owing to the robust bonding affinity of the 2-MP molecule with Pb^2+^ ions through its bidentate anchoring, which prevents moisture from effectively competing and disrupting the connection between the passivating molecules and the perovskite surface. Consequently, the reactivity between ambient water molecules and the perovskite film is effectively suppressed, leading to a significantly enhanced energy barrier for the hydration reaction pathway (Fig. 3i). These enhancements translated to a boosted PCE of 20.28%, with an inspiring *V*_OC_ of 1.18 V, while the PCE of the control device is 18.35%^58^.

In general, this intricate interplay of Lewis acid and base additives holds the potential for enhanced PSC performance through defect passivation. Nonetheless, gaining a comprehensive grasp of the intricate mechanisms underlying complete passivation remains a formidable task, primarily due to the multifunctional nature of certain Lewis acids/bases. The integration of systematic theoretical simulation and experimental verification is essential to achieve effective passivation in various perovskite fabrication processes.

#### The facilitation of charge extraction

**Fig. 4:**
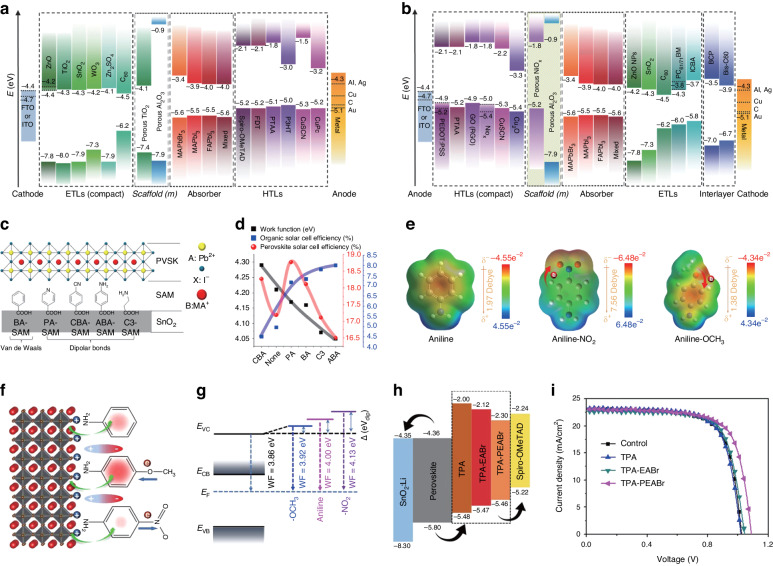
The interface modification between perovskite and charge transport layers. Energy level diagram of perovskites and charge transporting layer for **a** n–i–p and **b** p–i–n architecture PSCs. Reproduced with permission from ref. ^293^ Copyright 2018 Wiley-VCH. **c** The self-assembled monolayers between the SnO_2_ and perovskite film. (BA is benzoic acid, PA is 4-pyridine carboxylic acid, CBA is 4-cyanobenzoic acid, ABA is 4-aminobenzoic acid, and C3 is 3-propanoic acid). **d** The work function and corresponding PCE for perovskite after being treated by different self-assembled monolayers. Reproduced with permission from ref. ^63^ Copyright 2017 American Chemical Society. **e** Chemical structures and electrostatic potential mapping images of the three molecules. **f** Schematic diagram of the effect of coulomb force regulation and their surface dipole direction by different functional groups. **g** Schematic energy-level diagrams of control, −OCH_3_, aniline, and −NO_2_ molecule-treated CsPbBr_3_ films. Reproduced with permission from ref. ^64^ Copyright 2021 American Chemical Society. **h** The band alignment diagram. **i** J–V curves for PSCs treated with different molecules. Reproduced with permission from ref. ^65^ Copyright 2020 American Chemical Society

Enhanced charge extraction can be achieved through meticulously optimized energy level alignment, typically established at the interface between the electron transport layer (ETL)/perovskite or hole transport layer (HTL)/perovskite. Through the incorporation of an interfacial layer or the meticulous adjustment of either ETL or HTL band alignment, electron/hole transfer and extraction can be improved and *V*_OC_ can be enhanced as well. The band offsets between the ETL and perovskite, as well as between the HTL and perovskite, play a pivotal role in governing carrier recombination at the relative interfaces. In practical terms as Fig. 4a, b, achieving a band offset of approximately 0.2 eV becomes imperative to facilitate effective charge extraction at the ETL/perovskite and HTL/perovskite interfaces^59,60^. The energy level disparities between neighboring layers can be fine-tuned by means of interface engineering^61^.

##### Perovskite/ETL interface

Enhancing electron extraction and injection represents a crucial avenue for augmenting the efficiency of PSCs. These pivotal processes transpire at the interface of the ETL and the perovskite layer. Hence, the property of this interface wields significant influence over the whole device’s performance.

Rubidium bromide (RbBr) was employed to deposit onto the SnO_2_ surface. The introduction of RbBr was found to exert a transformative effect, resulting in the narrowing of SnO_2_’s bandgap from 3.58 to 3.34 eV. This modification led to a reduction in the energy barrier at the ETL/perovskite interface, thereby enhancing electronic contact between the two components. This pivotal alteration significantly contributed to the pronounced enhancement in overall device performance^62^.

Nonetheless, Yang et al. have introduced diverse functional groups onto the surface of SnO_2_ to establish a range of chemical interactions with the perovskite layer (Fig. 4c). Surprisingly, the performance of the perovskite solar cell devices deviates from the expected trend dictated by energy level alignment theory. This phenomenon underscored the pivotal role of chemical interactions as the predominant determinant of interfacial optoelectronic properties (Fig. 4d). Notably, the utilization of a self-assembled monolayer (SAM) composed of 4-pyridinecarboxylic acid yields the highest PCE, highlighting the profound impact of tailored chemical interactions on enhancing device performance^63^.

Therefore, in the process of choosing molecules for interface modification, it is imperative to consider not only the alteration in work function but also the ramifications of interfacial chemical interactions.

##### Perovskite/HTL interface

The role of HTL encompasses both electron blocking and hole transport functions. A crucial step within PSCs is the extraction of holes, which takes place at the interface between the HTL and the perovskite layer. Achieving highly efficient hole extraction at the HTL/perovskite interface holds significant potential for enhancing device performance.

Tang et al. explored the utilization of a series of self-assembled aniline molecules to modify the surface of CsPbBr_3_ films. By altering the functional groups at para-position which exhibited varying electronegativities such as electron-withdrawing −NO_2_ and electron-donating −OCH_3_, remarkable enhancements in photovoltaic parameters are observed (Fig. 4e). As depicted in Fig. 4f, the introduction of –OCH_3_ led to the greatest electron accumulation within the benzene ring, in contrast to –NO_2_ and pristine aniline. The discrepancy arose due to the charge transfer initiated by the disparity in electronegativities. This phenomenon enhanced the electrostatic force, thereby facilitating both hole transfer and hole extraction from the perovskite to the carbon electrode. Based on the analysis of ultraviolet photoelectron spectroscopy (UPS), the work functions for the control, −OCH_3_-, aniline-, and −NO_2_-sample are established as −3.86 eV, −3.92 eV, −4.00 eV, and −4.13 eV, respectively (Fig. 4g). Integrating insights from the UPS spectra depicting the valence band evolution, alongside the unaltered bandgap of 2.35 eV, the energy level diagram of CsPbBr_3_ PSCs post-treatment unveiled a perceptible transformation manifesting as a surface shift towards less n-type behavior in CsPbBr_3_ and an elevated energy level. Notably, this elevation proved advantageous for both hole extraction and the redirection of photogenerated electrons away from the interface. Particularly, a − OCH_3_-tailored all-inorganic CsPbBr_3_ solar cell achieved a PCE of 9.81%, accompanied by a remarkably improved *V*_OC_ of 1.632 V^64^.

Li et al. investigated the effect of N-((4-(N,N,N-triphenyl)phenyl)ethyl)ammonium bromide (TPA-PEABr) in the PSCs. This molecular, together with triphenylamine (TPA) and *N*-(2-(*N*,*N*,*N*-triphenyl)ethyl)ammonium bromide (TPA-EABr), was strategically introduced as an interface buffer layer via spin-coating onto the perovskite surface. The investigation unveiled that the highest occupied molecular orbital (HOMO) energy level of these TPA derivatives falls within the range of approximately −5.4 to −5.5 eV (Fig. 4h). Significantly, this energy level positioning places it between the perovskite layer and the HTL, effectively bridging the energy gap and contributing to an enhanced alignment of energy levels between these components. In contrast to the control devices, the PCE of PSCs treated by TPA remained nearly unchanged. However, an enhancement in PCE was observed from 16.69% to 17.40% when treated by TPA-EABr, and ultimately reaching an impressive 18.15% for the devices treated by TPA-PEABr. The enhanced performance stems from the surface passivation provided by TPA-PEABr, coupled with the refined alignment of energy levels achieved upon the incorporation of TPA-PEABr as the buffer layer^65^.

#### Tandem solar cells

**Fig. 5:**
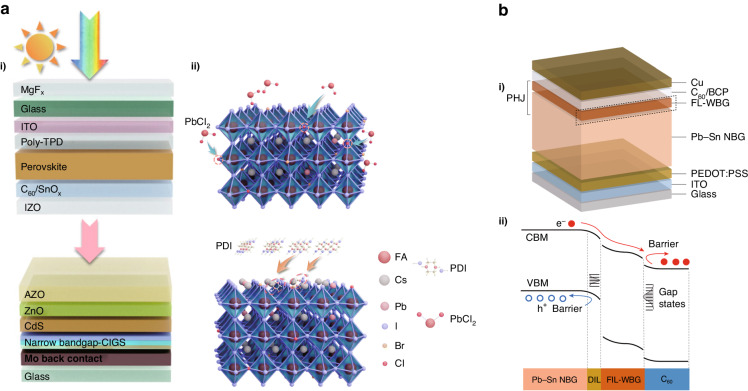
Perovskite tandem solar cells. **a** (i) Schematic of the 4-T PSC/CIGS tandem solar cell and (ii) the Cl bulk incorporation and PDI surface treatment. Reproduced with permission from ref. ^68^ Copyright Royal Society of Chemistry. **b** (i) The schematic structure and (ii) the energy diagram of Pb–Sn PSCs with a 3D/3D bilayer PHJ. Reproduced with permission from ref. ^66^ Copyright 2023 Springer Nature

Increasing the PCE of solar cells to its theoretical limit is the key to minimizing energy losses and improving cost-effectiveness. While the current PCE of single junction devices is not up to expectations, tandem solar cells with a wide bandgap absorber and a low bandgap absorber can maximize light utilization, resulting in a more desirable PCE. The perovskite with adjustable bandgap can be combined in tandem cells with both wide and low bandgap materials, such as perovskite/organic, perovskite/perovskite, perovskite/Si, perovskite/CIGS. Excitingly, the certified efficiency of perovskite/Si tandem cells has surpassed 33.7%, which is above the theoretical Shockley-Queisser limit (33%)^66^. However, the theoretical efficiency of the perovskite/Si tandem cell is much higher than that, and the main source of energy loss is the poor quality of the perovskite^67^. During cell preparation, perovskite is deposited directly onto the rough Si bottom-cell surface while the electrodes are deposited directly onto the perovskite. This makes obtaining high-quality perovskite difficult. Researchers have proposed the following strategies to improve the quality of perovskite: inserting a buffer layer to protect the perovskite, precursor solution engineering to improve crystalline growth, and passivating the perovskite surface to reduce defects. Similarly, the high roughness of the CIGS subcell surface is a major barrier to the preparation of high-quality uniform perovskite films. In addition to this, the unbalanced efficiency and bandgap mismatch between subcells limits the PCE that can be achieved. Liu et al.^68^ greatly improved the efficiency of perovskite with a bandgap of 1.67 eV achieving bandgap and *J*_SC_ matching with CIGS (*E*_g_ = 1.04 eV) through Cl native doping and piperidinium iodide (PDI) surface treatment of CsFAPb(IBr)_3_ (Fig. 5a). As a result, this PSC/CIGS tandem cell obtained the highest PCE of 28.4% to date. For perovskite/organic tandem cells, the low PCE of wide bandgap PSCs is the main reason hindering their development. In recent, Wang et al.^69^ reduced non-radiative complexation in wide-bandgap perovskite by a mixed cation (CF_3_-PEA^+^/EDA^2+^) passivation strategy. They achieved high *V*_OC_ (1.35 V) and FF (0.83), which resulted in a record PCE of 24.47% for the perovskite/organic tandem cell. The perovskite/ perovskite tandem cell has been given high expectations due to the lower cost of perovskite compared to the above materials. However, its performance is limited by the high trap density and Sn^2+^ oxidation brought by the narrow bandgap perovskite mixed with Sn/Pb. Tan et al. group reported an all-perovskite tandem cell with a 3D/3D bilayer perovskite heterojunction (Fig. 5b)^66^. This construction with a type II energy band structure at the interface of the perovskite/ETL suppressed interfacial nonradiative recombination and promoted charge extraction. This led to an increase in PCE to 23.8% for single-junction tin-lead perovskite and a maximum PCE of 28.5% for all-perovskite tandem cells. However, the high PCE of the tandem cell is based on high cost. Improving single-junction efficiency and matching between subcells to improve cost-effectiveness is essential.

In all, by leveraging the defect passivation of perovskite thin films, it is possible to attain high-quality thin films. Besides, conducting thorough optimizations of electrode materials, ETL, and HTL can significantly augment the overall photovoltaic performance of the device. Nevertheless, addressing the interface issue between the functional layers is essential to guarantee the efficiency of photovoltaic devices. Hence, qualified interface engineering holds the key to unlocking the full potential of perovskite photovoltaics, propelling the PCE of PSCs closer to its theoretical Shockley–Queisser limit^70^. Moreover, enhancing the light utilization rate within the device, for instance, by minimizing light reflection, can yield more significant improvements^71^. On the other hand, the concept of TSCs introduces a tangible avenue towards authentic third-generation thin-film photovoltaics, evading the confines of the traditional Shockley–Queisser single-junction limit. Presently, the zenith of achievable PCE in all-perovskite TSCs has ascended to an impressive 28%, while the pinnacle PCE in perovskite/silicon TSCs stands at a remarkable 33.7%^66^. Furthermore, the attainment of higher PCE is attainable through the enhancement of solar energy capture by stacking additional solar cells^72^. By implementing this comprehensive series of optimizations, a substantial improvement in the photovoltaic performance of the device is anticipated.

### The stability and improvement strategies

While PSCs have achieved remarkable success in terms of PCE, the significant challenge of ensuring their stability remains a substantial hurdle in the path toward industrialization. PSC device instability stems primarily from two overarching factors: internal issues and environmental influences. The internal factors encompass the structural stability of perovskite, notably addressing both its inherent stability and phase segregation. Meanwhile, environmental considerations encompass aspects such as humidity, oxygen, light, and thermal stability.

#### Intrinsic factors and strategies

**Fig. 6:**
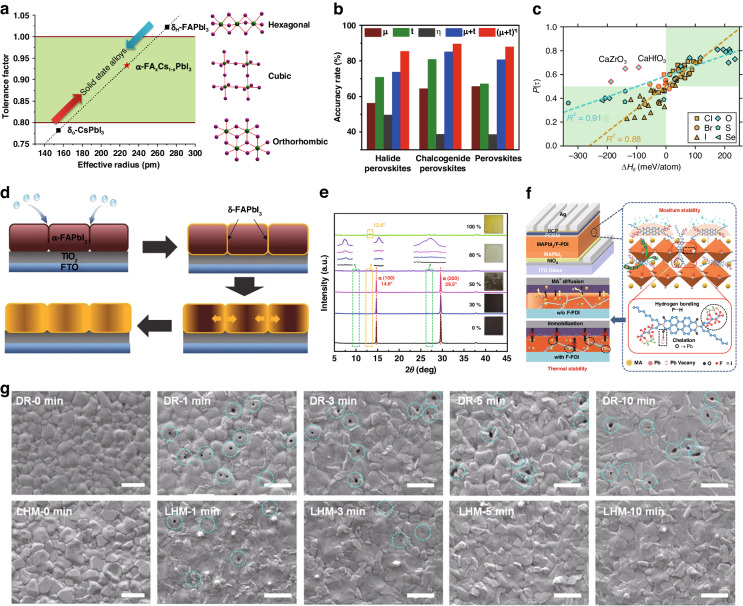
The intrinsic and humidity stability of perovskite. **a** Correlation between tolerance factor and structure of perovskite crystals. Reproduced with permission from ref. ^77^ Copyright Royal Society of Chemistry. **b** Accuracy rate for *μ*, *t*, *η*, (*μ* + *t*), and (*μ* + *t*)^η^ to forecast the relative stability of two perovskites. Reproduced with permission from ref. ^80^ Copyright 2017 American Chemical Society. **c** Comparison between P(t) and the decomposition enthalpy (∆*H*_d_) for 36 double perovskite halides. **d** Schematic diagram for the different periods in the degradation process. Reproduced with permission from ref. ^84^ Copyright 2018 Wiley-VCH. **e** The PXRD patterns of CsPbI_2_Br films stored under different humidity conditions and their corresponding photographs Reproduced with permission from ref. ^86^ Copyright 2022 American Chemical Society. **f** Device architecture of the PSCs. (Upper left) Schematic diagram of the interaction between F-PDI and perovskite (Right) Schematic diagram of thermal degradation for pristine perovskite and perovskite with F-PDI. (Lower left) Reproduced with permission from ref. ^89^ Copyright 2019 Wiley-VCH. **g** SEM images were recorded at different periods for the perovskite films annealed under dry N_2_ and low humidity (the scale bar is 1 µm). Reproduced with permission from ref. ^91^ Copyright 2021 Wiley-VCH

Although single-component perovskites have achieved good results, the thermal stability of MAPbI_3_ needs further enhancement. As for FAPbI_3_, despite its more suitable bandgap, the presence of an undesired phase transition poses challenges in maintaining the α phase with active photoelectric properties. Besides, organic-inorganic hybrid perovskites are commonly acknowledged to undergo irreversible decomposition at elevated temperatures, yielding organic halides, lead halides, and other volatile organic compounds. The degradation process results in the collapse of the 3D perovskite structure and the release of organic compounds, adversely affecting device performance. In contrast, CsPbI_3_ perovskite demonstrates superior intrinsic resistance to thermal stress, attributed to its exclusion of volatile and degradable components, unlike the volatile MA- and FA-based perovskites. Hence, the thermal stability of all-inorganic perovskite CsPbI_3_ is much better than organic-inorganic hybrid perovskite^73^. However, the complicated crystal phases (perovskite phase: α, β, γ phases; non-perovskite phase: δ phase) and their undesirable phase transition will hinder their industrial development. The predominant approach for tailoring optoelectronic properties and ensuring enduring stability in PSCs involves manipulating ions in the A, B, and X sites of a standard ABX_3_ perovskite framework. The structure of 3D perovskites can be predicted by the octahedral factor (*μ*) and Goldschmidt’s tolerance factor (*t*)^74^.

1 $$\mu =\frac{{R}_{B}}{{R}_{A}}$$

2 $$t=\frac{{r}_{A}+{r}_{X}}{\sqrt{2}({r}_{A}+{r}_{X})}$$

where *r*_i_ is the ionic radii of each ion (A, B, X). Research has revealed that the stable range for metal halide perovskite falls within 0.813 < *t* < 1.107 as well as 0.377 < *μ* < 0.895. And 0.8 < *t* < 1 could be conducive to maintaining the cubic perovskite structure^75,76^. Optimal structural stability is attained at *t* = 1, while any deviation from unity is likely to induce distortion in the BX_6_ octahedron. The relationship between the perovskite structure and the *t* is depicted in Fig. 6a. In the realm of inorganic-organic hybrid halide perovskite materials, an orthorhombic structure typically emerges when the *t* is below 0.8, while a cubic structure predominates in the range of 0.8 < t < 1. When t surpasses 1, a hexagonal structure tends to manifest. However, a larger A-cation yields the *t* value exceeding unity, giving rise to a layered perovskite arrangement, exemplified by the Ruddlesden–Popper (RP) phase. Tolerance factors below 0.7 yield non-perovskite structures^77^.

Under the above theoretical background, researchers have optimized the composition to enhance the performance of perovskite, making it exceedingly more suitable for practical applications. Owing to the relatively expansive FA^+^ ion (about 253 pm), the *t* of FAPbI_3_ slightly surpasses 1. Consequently, FAPbI_3_ readily assumes the δ phase at standard room temperature conditions. Hence, there arises a need to diminish the tolerance factor of FAPbI_3_ while concurrently elevating the activation energy barrier for the phase transition from α to δ phase. Li et al. tried to alloy FAPbI_3_ and CsPbI_3_ (*t* ≈ 0.8). This alloying approach effectively reduced the required treatment temperature for the δ to α phase transition, lowering it from 165 °C for pure FAPbI_3_ to below 100 °C for FA_1-x_Cs_x_PbI_3_ Consequently, this technique enables precise adjustment of the effective tolerance factor and subsequently bolsters the stability of the α-phase with photoactivity in the composite FA_1–x_Cs_x_PbI_3_ alloys^78^. Besides Cs^+^, the mixture of MA^+^ and FA^+^, resulting in the creation of FA_1−x_MA_x_PbI_3_, notably enhanced the stability of the α phase. Nazeeruddin et al. made a discovery regarding the enhancement of MAPbI_3_ film properties. Through the addition of 10% FA^+^, they observed a remarkable improvement in both crystallization and compositional uniformity. This enhancement was attributed to the self-organization of the MAPbI_3_ film into a stable “quasi-cubic” phase even at room temperature^79^.

Furthermore, the thermal stability for 138 cubic perovskite material was thoroughly investigated through a comprehensive analysis of their decomposition enthalpies (∆*H*_*D*_), employing first-principles density functional theory (DFT) calculations. This endeavor yielded a noteworthy discovery: a linear correlation between ∆*H*_*D*_ and the product of (*(t* + *μ*)^*η*^, where *η* represents the atomic packing fraction. Serving to be an effective thermodynamic stability descriptor, the (*t* + *μ*)^*η*^ combination can accurately predict stabilities in halide (chalcogenide) perovskite variants with a commendable precision rate of 86% (90%) (Fig. 6b)^80^.

Nonetheless, Scheffer et al. uncovered an inadequacy in the predictive precision of *t*. Their investigation revealed a significant margin of error, nearly one-quarter, in assessing the crystal structure of perovskites by *t*, particularly in cases involving materials with heavier halides^81^. Hence, the formula of tolerance factor was modified to make it more applicable for materials discovery. The new formula for the tolerance factor is presented below:

3 $$\tau =\frac{{r}_{X}}{{r}_{B}}-{n}_{A}\left({n}_{A}-\frac{{r}_{A}/{r}_{B}}{{In}({r}_{A}/{r}_{B})}\right)$$

where *n*_A_ is the oxidation state of A. When *r*_A_ > *r*_B_, and *τ* < 4.18, this material exhibits the perovskite phase. The agreement between the observed values of *t* and the calculated stability is apparent in 64 out of the 73 materials investigated. Notably, the probabilities generated through classification using t exhibit a linear correlation with the ∆*H*_*d*_, which proved the value of the monotonic behavior between *τ* and *P(τ)*, where the *P(τ)* is *τ*-based probability of being perovskite (Fig. 6c). Using this updated formula, the experimental dataset achieved an impressive overall accuracy of 92%. Hence, drawing upon both Eqs. (2) and (3), it becomes feasible to precisely anticipate the crystalline arrangement of a perovskite composition. In the pursuit of cutting-edge photovoltaic applications, the ability to finely manipulate structural attributes holds paramount importance for attaining the pinnacle of efficiency and stability in advanced PSCs.

In the culmination of these endeavors, the strategic intermingling of cations characterized by distinct steric sizes (such as Cs, MA, FA) at the A site, or variations in anions (I, Br, Cl) at the X site, introduces a modifiable effective ionic size. This nuanced adjustment to the tolerance factor (*t*) brings it within a stable range. These empirical principles have provided invaluable guidance in achieving the stabilization of perovskites and in embarking on the exploration of newly emerging and stable perovskites.

#### External factors and strategies

Alongside intrinsic factors, external environmental factors also wield significant influence over the performance of PSC devices. By encapsulation, PSCs can be effectively prevented from the environmental atmosphere. However, the challenge of realizing an ideal encapsulation necessitates an exploration of the stability of PSCs influenced by environmental factors. The key destabilizing factors for PSCs encompass humidity, oxygen, temperature, and light.

##### Humidity stability

Based on earlier experimental results, unencapsulated cells underwent degradation in several hundred hours when exposed to air under humidity exceeding 50%^82,83^. The phase transition unfolds in a gradual manner, advancing from the grain boundaries toward the interiors of the grains. These dynamic mechanisms are visually depicted in Fig. 6d^84^. As degradation persists, the phase transition extends further into the grain interiors, eventually converting adjacent grains into the non-perovskite phase. Within perovskite crystals, water molecules establish strong hydrogen bonds with organic components. This interaction serves to diminish the bond strength between the organic component and the PbI_6_ octahedron, facilitating a more rapid deprotonation of the organic cation. Additionally, water contributes to the protonation of iodide, leading to the formation of volatile HI. Consequently, this process leaves behind PbI_2_ as a residue of decomposition^85^. Figure 6e revealed that α-CsPbI_2_Br transit into δ-CsPbI_2_Br at first and decomposed into PbI_2_, which was influenced by relative humidity level and storage time^86^. To bolster perovskite’s humidity stability, two commonly employed strategies are dimension engineering and surface modification.

Dimension engineering is commonly employed to enhance humidity stability and strengthen the activation energy barrier. Two-dimensional (2D) perovskites demonstrate notable stability but exhibit limited PCE. To concurrently boost both the PCE and stability, a new era of mixed-dimensional (MD) perovskite has emerged. Zheng et al. orchestrated MD perovskite by incorporating HOCH_2_CH_2_NH_3_I (EAI) into the (FAPbI_3_)_0.85_(MAPbBr_3_)_0.15_ 3D perovskite. Upon enduring exposure to approximately 50% relative humidity for more than 1700 h, unencapsulated devices preserved approximately 85% of their initial PCE when incorporated with EAI. However, the 3D perovskite film degrades after 400 hours of storage and swiftly transforms into yellow PbI_2_ within a brief period in a humid environment. This marked improvement is ascribed to the exceptional crystal structure and surface morphology^87^. Li et al. engineered a novel heterostructure, termed Localized Dion-Jacobson (DJ) 2D–3D Heterostructures (L2D–3DH), by selectively growing the DJ phase on 3D perovskite films. This selective growth, achieved through post-treatment with divalent organic spacer cations (1,4-butanediamine iodide), enhances grain boundary passivation and prevents moisture penetration. Unlike conventional 2D–3D composites, this design minimally hinders charge extraction due to exposed 3D regions, eliminating the need for precise orientation control. PSCs based on L2D–3DH demonstrated remarkable advancements, reaching a PCE of 20.1%, slightly surpassing pure 3D-based PSCs (19.7%). Enhanced PCE is attributed to DJ 2D plate-induced grain boundary passivation, reducing trap density and non-radiative recombination. Impressively, L2D–3DH-based PSCs exhibit extended stability under high moisture without full 2D film coverage. Initial PCE of 86% reserved with unencapsulated L2D–3DH-based PSCs after 1300 hours under 70% RH, outperforming 3D counterparts at 56%. Under heat and high humidity stress, an initial PCE of 75% was reserved with L2D–3DH-based PSCs after 200 hours of continuous aging at 80 °C and 70% RH^88^.

Passivating grain boundaries and modifying the surface of perovskite can also mitigate moisture-induced degradation of the perovskite layer. Yang et al. incorporated N, N’-bis-(1,1,1,2,2,3,3,4,4-nonafluorododecan-6-yl)-perylenediimide (F-PDI) into perovskite, resulting in defect passivation and the formation of a hydrophobic structure, and thereby remarkable enhancing photovoltaic performance and device stability. The carbonyl groups in F-PDI chelated with uncoordinated Pb^2+^, which led to defects passivation at grain boundaries and the perovskite surface. The F-PDI molecules with strong conductivity facilitated charge transfer across grain boundaries, enhancing photovoltaic properties. Additionally, hydrogen bonding between fluorine groups and MA could fix the MA^+^ ion (Fig. 6f). Notably, the inherent hydrophobicity of F-PDI shielded perovskite from moisture, substantially bolstering humidity resistance in PSCs^89^. Zhan et al. introduced a versatile self-encapsulation method for crystal growth using polymer-assisted bottom-up dynamic diffusion. A polymer scaffold formed during nucleation and is subsequently etched by the anti-solvent, guiding perovskite growth. This approach balanced nucleation density and growth rate, enhancing crystalline quality by preventing excessive precursor-polymer interaction. The dynamic diffusion involved polymers in nucleation and growth, resulting in controlled nucleation and phase separation-driven encapsulation. The distribution of polymers such as polyethylene glycol (PEG) or polystyrene (PS) at surface, grain boundaries, or buried interfaces sequentially passivated defects, aligned energy bands, and aided carrier transportation, yielding PSCs with high *V*_OC_ (1.15 V), FF (80.72%), and PCE (22.90%). Moreover, self-encapsulated PSCs exhibited remarkable environmental stability, with negligible decomposition and 90% PCE retention after 90 days under 30-50% humidity in ambient air^90^.

While perovskite degradation transpires in high humidity conditions, empirical investigations have demonstrated that perovskite films can self-heal defects through exposure to mild humidity. Figure 6g portrays the dynamic transformations of film morphology during the annealing process under dry (DR) and low humidity (LHM) atmospheres (30-40% relative humidity). Initially, the film exhibited an uneven morphology with small and irregular grains (DR-0 min). Once exposed to LHM, the grains grew larger with better uniformity (LHM-0 min), signifying a moisture-induced phase transition. Throughout annealing, both atmospheric conditions led to grain size augmentation and textured surfaces, attributed to perovskite crystal growth. Notably, for the DR scenario, annealing resulted in numerous pinholes (DR-1 min), persisting in the final DR-perovskite film. In contrast, LHM annealing produced fewer pinholes (LHM-1 min), subsequently diminishing during the process. The ex-situ SEM observations validated the gradual healing of pinholes and defects in FA-based perovskite films directly through humidity-annealing. In device application, films prepared under LHM exhibit optimal PCE, primarily due to enhanced *V*_OC_ and FF. Further optoelectronic analyses confirmed that improved device performance stems from reduced defects in the film. These experimental findings underscored that humidity operated as a two-edged sword. Reasonable humidity tuning can enhance the performance of PSCs significantly^91^.

##### Oxygen stability

**Fig. 7:**
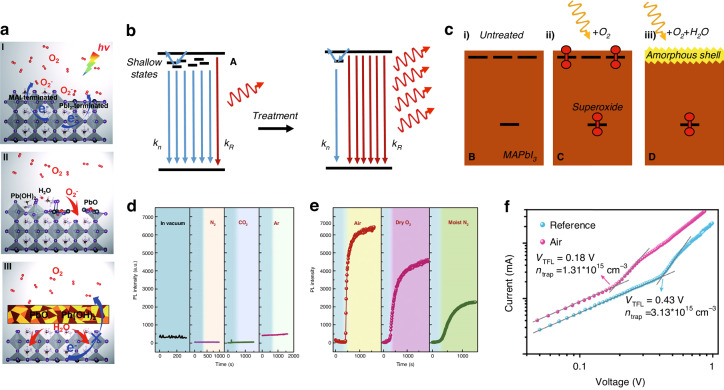
The oxygen stability of perovskite. **a** Diagram illustration of the photo-oxidative degradation mechanism of the MAPbI_3_ (001) surface. Reproduced with permission from ref. ^97^ Copyright Royal Society of Chemistry. **b** Schematic depicting the shift from non-radiative recombination (k_n_) dominance due to the presence of shallow surface states, to radiative-dominant recombination (k_R_) following the removal of these states through treatment. Untreated MAPbI_3_ film **c** (i) comprising non-radiative trap states which are passivated upon MAPbI_3_ exposed to (ii) light and oxygen and (iii) light, oxygen, and water. Reproduced with permission from ref. ^102^ Copyright 2017 Elsevier Ltd. **d** PL intensity as a function of time in vacuum and on exposure to dry N_2_, dry CO_2_, and dry Ar. **e** PL intensity as a function of time on exposure to air, dry O_2_, and moist N_2_. Reproduced with permission from ref. ^103^ Copyright 2016 American Association for Advancement of Science. **f** Characteristics of devices utilizing both the reference film and the air-CsPbI_2_Br film. Dark current-voltage measurements were conducted on electron-only devices. Reproduced with permission from ref. ^104^ Copyright 2019 American Chemical Society

Some experiments have shown that metal halide perovskites could exhibit relative stability to oxygen when kept in the dark, suggesting their fair stability in the ground state^92,93^. However, upon light exposure, the MAPbI_3_ perovskite layer undergoes rapid degradation. Oxygen can trigger the degradation of perovskite film in specific circumstances. As shown in Fig. 7a, there is a schematic illustration of the photo-oxidative degradation process of the MAPbI_3_ (001) surface. Step I involves the interaction between the O_2_ near the surface of MAPbI_3_ and the photo-excited electrons from MAPbI_3_. This interaction resulted in the formation of superoxide (O_2_^−^). For Step II, these O_2_^−^ undergo a reaction with [CH_3_NH_3_]^+^ ions and Pb atoms, leading to the production of H_2_O and Pb(OH)_2_ on the surface terminated with MAI, thereby exposing the underlying MAI-terminated surface. In Step III, the oxidation products generated in the previous steps restrain the oxidation of the inner MAPbI_3_. Next, the water molecules contribute to the hydration of the inner perovskite structure. As a result of this hydration process, the inner perovskite gradually disintegrates, leading to the breakdown of the entire perovskite structure over time^94–97^. What’s more, oxygen has the potential to oxidize metal oxide charge transport materials, particularly TiO_2_. TiO_2_ is notably susceptible to reacting with ambient oxygen, resulting in the formation of superoxide, which then contributes to the oxidative degradation of perovskite^98,99^.

To diminish the adverse effect of oxygen on device performance, the incorporation of a 2D layer proves to be a notably effective approach. Shao et al. demonstrated that the incorporation of a small amount of 2D tin perovskite into a 3D tin perovskite led to an enhancement in the crystallinity of 3D FASnI_3_. The extended arrangement of crystal planes has significantly fortified the resistance ability and structural integrity of the perovskite framework, concurrently mitigating tin vacancies and minimizing background carrier density. The substantial crystalline quality and preferential orientation significantly underlie the improved solar cell performance. Moreover, device stability under non-encapsulated ambient conditions (humidity ~20%, temperature ~20 °C) was assessed. The device employing a hybrid 2D/3D structure exhibited significantly greater stability in comparison to its pure 3D counterpart. Following a 76-hour exposure to ambient air, the pure 3D perovskite device experienced a complete failure. Conversely, the 2D/3D composite device demonstrated remarkable resistance by preserving an impressive 59% of its initial PCE. Further XRD measurements were performed on perovskite samples stored in nitrogen atmosphere and ambient air. The 3D and 2D/3D samples decomposed slightly after 6 hours in an inert atmosphere. Notably, the 3D sample exhibited much quicker chemical degradation than the 2D/3D sample when exposed to ambient conditions. This enhanced ambient stability of the 2D/3D-based device was likely attributed to heightened resistance ability to oxygen and moisture, stemming from improved crystallinity and higher perovskite film hydrophobicity^100^.

While the presence of oxygen can substantially weaken the chemical stability of perovskite materials due to reactions with protonated organic cations like MA^+^ and FA^+^, it’s important to highlight that leveraging oxygen for defect passivation has also arisen as a potent and convenient strategy in suppressing nonradiative recombination processes and bolstering the photovoltaic performance of PSCs by strongly interacted with halide vacancies located on the perovskite surface^101^. The mechanism of photo brightening is given in Fig. 7b. After being treated by light, O_2_, and humidity, the density of the shallow state below the conduction band will decrease, leading to the increase of the radiative bimolecular recombination (k_R_) and decrease of non-radiative bimolecular component (k_n_), which significantly enhance the photoluminescence quantum yield (PLQY). The detailed passivation mechanism is as follows: Initially, an untreated MAPbI_3_ sample with surface states (Fig. 7cii) was subjected to illumination in the presence of O_2_. This illumination-induced process made a reduction to the density of surface states as well as triggered photo brightening, as depicted in Fig. 7cii). The mechanism behind this phenomenon involved the formation of passivating superoxide species. Importantly, the transformation was reversible and occurred in several hours when subsequently shielded from light. Subsequently, an untreated sample was momently subject to both illumination and a combination of H_2_O and O_2_ (Fig. 7ciii). In this case, the reduction in the density of shallow states became much more pronounced owing to the complete elimination of surface states. This elimination resulted from the formation of a nanometer-thin amorphous shell composed of inert degradation products. This process is nearly irreversible, and the shell of degraded material effectively serves as a containment barrier by which oxygen species can just tardily escape from the film. These treatments effectively reduce ion migration, as oxygen molecules occupy iodide vacancy sites integral to the ion migration process. Moreover, the presence of the degraded shell introduced a partial impediment to ionic transport within the intergrain regions^102^. Fang et al. explored the change in PL intensity over time for a single crystal while exposed to various gas atmospheres during illumination (Fig. 7d, e). The PL intensity of the crystal remains unaffected when exposed to dry N_2_, CO_2_, or Ar. However, a significant and swift increase in PL intensity occurred in the presence of air, dry O_2_, and humidified N_2_. It’s noteworthy that the most rapid and pronounced enhancement in PL intensity was observed when the crystal was exposed to air. In contrast, the recovery of PL intensity was notably slower in the case of dry O_2_ and humidified N_2_. These observations highlighted the role of molecular properties, specifically those of O_2_ and H_2_O, in driving the PL enhancement, which is consistent with the mechanism mentioned above^103^. Unlike the conventional method of oxygen molecule passivation via surface physisorption on perovskites, Liu et al. discovered that individual oxygen atoms offer superior passivation because of the stronger interaction with perovskite. Crucial to attaining this objective is dry-air processing, dissociating O_2_ into O during annealing. O-passivated inorganic halide PSCs exhibited less density of defects, higher PV performance and improved air stability compared to O_2_-passivated devices, as shown in Fig. 7f^104^.

##### Light stability

Although environmental variables like oxygen and humidity can be addressed through encapsulation, it’s imperative for the solar cell to maintain its light stability over extended periods. Light-induced phenomena significantly influence perovskite materials, primarily manifesting as halide segregation, ion migration, and triggering irreversible photochemical reactions^105–107^.

**Fig. 8:**
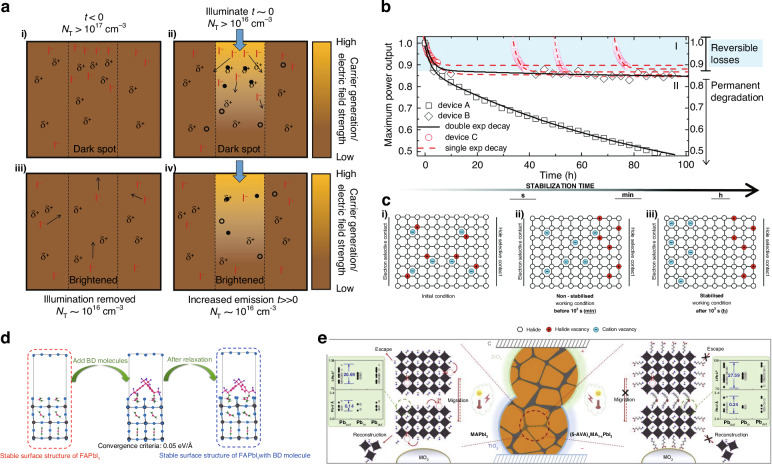
The light stability of perovskite. **a** (i) The density of traps within a ‘dark spot’ is notably elevated, accompanied by an excess of iodide ions at first. (ii) Upon exposure to light, electrons rapidly occupy traps, generating an electric field that prompts the migration of iodide away from the illuminated area, subsequently occupying the vacant positions. (iii) The system ultimately attains a stable emission output, accompanied by a diminished trap density and iodide concentration within the illuminated area. (iv) After the removal of illumination, concentration gradients may facilitate the return of some iodide back into the dark spot before eventually establishing a new equilibrium with a redistributed iodide profile. Reproduced with permission from ref. ^107^ Copyright 2016 Springer Nature. **b** Maximum power output tracking was conducted on three identically prepared PSCs, designated as devices A, B, and C, while exposed to UV-filtered 1 Sun equivalent light. Devices A and B were continuously monitored for more than 100 h, whereas Device C underwent cyclic tracking four times, with each tracking session lasting 5 h, interspersed with periods of being kept in the dark at an open circuit. Schematic illustrations were employed to visualize the evolution of ion distribution within the perovskite layer situated between the electron and hole selective contacts during the operational conditions of the solar cells: **c** (i) initial conditions, (ii) non-stabilized conditions in several minutes, and (iii) the stabilized condition in several hours. Reproduced with permission from ref. ^109^ Copyright Royal Society of Chemistry. **d** Theoretical simulation involving the incorporation of BD molecules into FA perovskites—illustrating the procedure for creating a stable surface structure of FAPbI_3_ with BD molecules. Reproduced with permission from ref. ^114^ Copyright 2023 Elsevier Ltd. **e** Illustrations depicting the structural configurations of MAPbI_3_ and (5-AVA)_x_MA_1-x_PbI_3_ within triple-mesoscopic layers, along with the mechanisms of material decomposition and ionic migration triggered by the combined influence of light, heat, and electronic bias. The green insets offer details regarding the Pb-I bond lengths and I-Pb-I bond angles within MA^+^-terminated slabs (left) and 5-AVA^+^-terminated slabs (right), respectively. Reproduced with permission from ref. ^115^ Copyright 2020 Elsevier Ltd

When subjected to AM 1.5 G solar simulator illumination, the photoluminescence intensity of MAPbI_3_ undergoes a strong enhancement in the beginning, accompanied by a drastic reduction in trap density under the influence of light^106,108^. This reduction translates into an augmented photovoltage within the device. The heightened photoluminescence intensity can be ascribed to the migration of I-species away from the irradiated zone. During the film formation process, specific regions exhibited a higher concentration of electron traps, likely stemming from iodine vacancies and associated interstitial iodine ions, which predominantly existed at the surface and grain boundaries, as shown in Fig. 8ai. Upon exposure to light, a notable density of light-excited electrons and holes emerges, most concentrated at the surface and gradually declining through the film. A considerable number of these light-excited electrons tend to be trapped, particularly in proximity to surfaces (Fig. 8aii). Trap filling perturbs the system, generating an electric field that triggers iodide migration. This migration leads to trapping annihilation through various mechanisms, including coulomb repulsion between unscreened iodide ions currently, space charge separation due to surface-trapped electrons and diffused holes, and alterations in surface band bending under illumination. The resultant induced migration facilitates the movement of numerous mobile iodides to occupy the vacancies, ultimately reducing the density of vacancies and interstitials (Fig. 8aiii). Once removing the light, the profile of light-excited components dissipates, leaving some residual traps. This residual state allows for gradual lateral or vertical migration of iodides to establish a new equilibrium over time (Fig. 8aiv), resulting in a partial reversibility^107^.

When considering the deterioration caused by light exposure in photosensitive materials, it becomes crucial to take into account irreversible chemical reactions triggered by light. This process, known as photoinduced degradation, comprises two distinct phases: the first is a rapid degradation process that can be reversed, which is referred to as regime I), while the second is a slower degradation process that is irreversible (referred to as regime II), as shown in Fig. 8b. Domanski et al. conducted an extensive investigation involving the continuous monitoring of the maximum power output from three identical devices labeled as A, B, and C. Devices A and B underwent continuous monitoring for a duration exceeding 100 hours. Notably, device A exhibited a relatively unstable performance, while device B owned good stability. It’s worth highlighting that both devices shared an identical time constant during the decay regime I. To distinguish and study regime I independently from the following degradation, specific measures were taken for device C. In this regard, the maximum power point tracking (MPPT) for device C was intentionally paused after just 5 h of operation. Subsequently, the tracking was periodically re-initiated following periods of rest in darkness, with the duration of these resting periods being intentionally varied.

In relation to regime I, it is important to notice that both halide and cation vacancies possess mobility within the material, although cation vacancies exhibited slower mobility compared to halide vacancies. The distribution of these vacancies within the perovskite layer significantly influences the extraction of charges and consequently impacts the overall PCE of the device. Figure 8ci–iii illustrated the arrangement of halide lattice within the perovskite with ionic vacancies. The initial scenario (Fig. 8ci) depicts a balanced presence of anion and cation vacancies, randomly dispersed throughout the perovskite lattice (Fig. 8cii). Up to 100 seconds (equivalent to minutes), following the initiation of light exposure and the transition to the MPPT, halide vacancies migrate, forming a Debye layer at the interface with the hole-selective contact, while cation vacancies, with comparatively limited mobility, remain behind (Fig. 8ciii). As the timeframe extends beyond 1000 seconds (equivalent to hours), cation vacancies accumulate, forming an additional Debye layer at the interface with the electron-selective contact. This accumulation of cation vacancies impedes the extraction of charges from the device, contributing to the loss of efficiency. Consequently, under realistic operational conditions, the gradual migration of ions emerges as the underlying cause of reversible losses within the device over hours. When the device is allowed to recuperate in darkness for several hours, the distribution of ionic vacancies reverts back to its initial state^109^.

In the context of regime II, the irreversible process can be attributed to a chemical degradation reaction. Following an extended period of exposure to white light, the MAPbI_3_ film undergoes a transformation into MAI and PbI_2_. Over the course of 6 hours, PbI_2_ is liberated and subsequently disintegrates into Pb and I_2_. As the duration of light exposure reaches 24 hours, the decomposition process reaches saturation, establishing a self-limiting mechanism. However, these degradation products induce a bending of the band at the interface, introducing a constraint on carrier transport^110,111^.

When subjected to ultraviolet light, the N-H bonds within the perovskite lattice dissociate, leading to the generation of CH_3_NH_2_ and H_2_. Following a period of 2 hours, metallic Pb emerges as a result of decomposition, which progresses and eventually reaches a state of saturation. This intricate process of degradation under ultraviolet irradiation further underscores the dynamic behavior of the MAPbI_3_ film, influencing its structural integrity and performance characteristics^112^.

To address the challenge of prolonged instability, researchers are consistently making dedicated endeavors. Liu et al. have introduced a covalent bonding strategy utilizing bis-diazirine (BD) molecules to form robust covalent bonds with the organic cations in perovskite materials, which could crosslink aliphatic organic molecules which contain C–H, O–H or N–H bonds via high temperatures or UV light to activation of diazirine groups^113^. What’s more, the separation between the binding sites of BD molecules (9.20 Å) closely corresponds to the lattice size of FA perovskite (9.01 Å). Both experimental findings and ab initio simulations validate the remarkable effectiveness of BD molecules in firmly immobilizing these organic cations (Fig. 8d). As a result, the thermal, illumination, and electrical bias resistance properties of perovskites are significantly enhanced. This advancement has resulted in the achievement of exceptionally efficient PSCs, boasting a remarkable efficiency of 24.36%. Notably, these ultra-stable PSCs maintain 98.6% of their initial efficiency even after undergoing 1000 h of operational testing^114^. Han et al. have discerned that the primary cause of deterioration in MAPbI_3_ perovskite lay in the liberation of MAI at grain boundaries within an exposed area or the crystal’s reshaping within confined spaces (Fig. 8e). Furthermore, irreversible long-distance ionic migration was induced by the combined influences of light, heat, and electrical bias. By fortifying the grain boundaries with a bifunctional organic molecule, 5-ammoniumvaleric acid (5-AVA) iodide, the crystalline structure of MAPbI_3_ can be fixed on a nanoscale. Consequently, the disintegration or reshaping of the crystal was suppressed, and the ionic migration became reversible. This method provided a dependable way to meet IEC61215:2016 stability requirements for PSCs. Remarkably, a printable PSC embedded with (5-AVA)_x_MA_1-x_PbI_3_ has demonstrated its endurance, functioning for over 9000 h at a maximum power point of 55 °C ± 5 °C, with no discernible degradation^115^.

##### Thermal stability

**Fig. 9:**
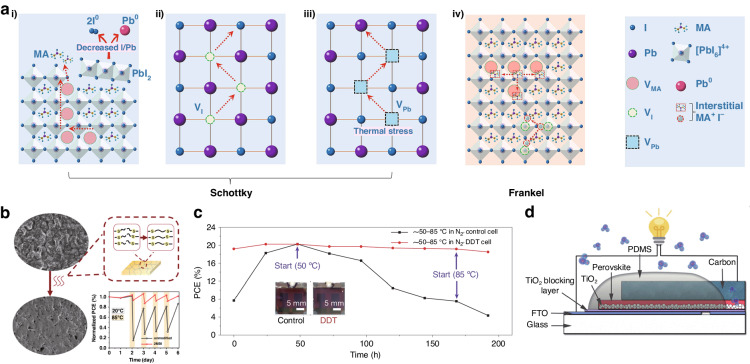
The thermal stability of perovskite. Illustration depicting ion migration and defect types in MAPbI_3_ following heating at 85 °C. Schottky defects: vacancy defects and migration of **a** (i) MA^+^, (ii) I^−^, (iii) Pb^2+^, and (iv) Frankel defects. Reproduced with permission from ref. ^120^ Copyright 2023 Elsevier Ltd. **b** Top-view SEM images: 2MBI-modified perovskites pre/post thermal degradation after 3 days storage and normalized PCE of devices across multiple heat treatment cycles. Reproduced with permission from ref. ^125^ Copyright 2022 American Chemical Society. **c** Performance evolution of the highly stable control and DDT-treated devices during thermal stress over 144 h. Insets: photographs of the control sample (left) and DDT-treated sample (right). Reproduced with permission from ref. ^131^ Copyright 2022 Springer Nature. **d** Schematic representation of the encapsulated device. Reproduced with permission from ref. ^136^ Copyright Royal Society of Chemistry

Given the necessity of high-temperature both in annealing for perovskite film and subsequent module encapsulation, coupled with the requirement for long-term stability at 85 °C for solar cells, enhancing the thermal stability of PSCs becomes imperative for their successful industrial implementation^116,117^. The exceptional light-harvesting capabilities of MAPbI_3_ progressively diminish with time as it transforms into PbI_2_ after the escape of MAI^118^. Zhu et al. depicted defect types and ion migration in inverted MAPbI_3_ PSCs with a schematic diagram (Fig. 9ai-iv). Primary Schottky defects include MA vacancies (V_MA_) and I vacancies (V_I_), while vacancy defects for Pb^2+^ are less owing to high energy barriers for their formation. Consequently, ion migration, particularly of MA^+^ and I^−^, is probable at room temperature, while Pb^2+^ migration requires thermal excitation. This migratory process intensifies at 85 °C, leading to the formation of PbI_2_ as MA^+^ ions escape. Additionally, reducing the I/Pb ratio on the MAPbI_3_ surface results in the emergence of metallic Pb^0^ defects (Fig. 9ai), and Pb ion migration is observed during continuous thermal aging (Fig. 9aiii)) Schottky defects often coincide with Frenkel defects as interstitial ions pair with vacancies (Fig. 9aiv)^119^. These ion migrations and defect accumulations at elevated temperatures contribute to structural changes in MAPbI_3_ perovskite materials, leading to device degradation^120^. What’s more, perovskite films can also degrade at lower temperatures over extended durations due to the volatilization of halide species and the organic cation, particularly when MA-containing compounds are involved^117^. As such, there is a pressing requirement to enhance the thermal stability of organic/inorganic hybrid perovskites.

Research has demonstrated that utilizing a blend of MAI and FAI in films created through a two-step deposition method, with MAI content below 20%, contributed to the trigonal phase maintaining its structural integrity across the studied temperature range (25 to 250 °C)^14^. Moreover, the slight introduction of MAPbBr_3_ to MAPbI_n_Br_3–n_ can enhance both PCE and thermal stability^121^. Besides crystallization optimization, dimension engineering or surface modification can also improve the thermal stability of perovskite^122–124^. For example, the application of a conjugated sulfide known as 2-mercaptobenzimidazole (2MBI) yielded remarkable enhancements in the PV characteristics and thermal stability of perovskite. During thermal processing, 2MBI formed interconnections on the perovskite surface, thereby facilitating the movement of charges, curbing the release of volatile components, and orchestrating the rearrangement of surface perovskite crystals. The PSCs modified by 2MBI attained a PCE of 21.7%, maintaining consistently impressive yield even while undergoing and following exposure to a temperature of 85 °C (Fig. 9b). In contrast, unmodified PSCs experienced significant degradation under similar conditions. Furthermore, unencapsulated devices, following thermal stress, maintained more than 98% of their initial efficiency after a 40-day storage period under ambient environmental conditions^125^.

Heightened temperatures not only lead to the degradation of perovskite but also affect the performance of the charge transport layer. Charge transport layers composed of inorganic metal oxides, such as TiO_2_ and NiO_x_, typically exhibit robust thermal stability owing to their high decomposition temperatures. However, in comparison, the organic charge transport layer’s thermal stability significantly lags behind that of its inorganic charge transporting layers^126–128^. For instance, iodine migration into Spiro-OMeTAD was observed when subjected to a 50-hour thermal treatment at 85 °C in an argon environment. This migration led to a reduction in oxidized Spiro-OMeTAD and subsequently lowered the conductivity of the transport layer^129^. To enhance the stability of Spiro-OMeTAD, additive engineering has proved to be an effective strategy. Based on the fundamental requirement of introducing a lithium compound (LiTFSI) for chemical doping to achieve satisfactory conductivity and efficient hole extraction in spiro-OMeTAD, Liu et al. incorporated an economical alkylthiol additive (1-dodecanethiol, DDT) into the spiro-OMeTAD HTL. Through the inclusion of DDT, a more effective and finely controlled doping procedure emerges, considerably reducing the duration of doping. This advancement empowered the HTL to attain comparable performance levels even before exposure to air activation. The synergy between DDT and LiTFSI enhanced the dopant concentration within the bulk of HTL. Consequently, it diminished dopant accumulation at interfaces and bolstered the overall structural resistance ability of HTL when subjected to conditions such as moisture, heat, and light exposure. Impressively, these devices exhibited remarkable durability, retaining 90% of their peak performance over a continuous 1000-h illumination period. The study delved into the effect of DDT on thermal stability, conducted through thermal stress experiments on unencapsulated PSCs. Notably, Fig. 9c illustrated a substantial increase of up to 63% in peak PCE at 50 °C after 120-h for the finest control device, which resulted from a drastic reduction in trap density under the influence of light^130^. In contrast, the most superior DDT-treated device displayed exceptional performance retention, up to 95%, during the same measurement interval. Subsequent evaluation at an elevated temperature of 85 °C revealed a speedier 16% deterioration for the control device after 24 h, while the DDT-treated device exhibited a more gradual decline, with only an additional 5% reduction^131^. Some other strategies have also been proposed to improve the thermal stability such as interface engineering or synthesis of new HTL materials^132–135^.

Additionally, through the adept application of suitable encapsulation methodologies, PSCs can endure even the most challenging environmental conditions (Fig. 9d). To delve deeper into the intricate chemistry of perovskites within operational PSCs, it is imperative to establish stringent encapsulation procedures that effectively shield against the impact of ambient air^136–138^.

To enhance the stability of PSCs, a comprehensive strategy should be implemented, incorporating a range of improvement measures. Initially, the optimization of materials and the fine-tuning of the perovskite formula are pursued to fortify its long-term stability. Subsequently, state-of-the-art encapsulation technology is employed, utilizing moisture-resistant, anti-oxidative, and UV-resistant materials to alleviate the impact of the external environment on the cell. Within the device structure, interface engineering is deployed to enhance interactions between layers, minimizing interface defects and elevating overall stability and performance. The use of appropriate ion migration inhibitors prevents the uncontrolled movement of charge carriers within the device, thereby retarding the aging process. Finally, the continual refinement of the production process ensures heightened production consistency, guaranteeing the stability and performance of each individual solar cell. These integrated enhancements collectively contribute to an augmented overall stability of PSCs.

### Large-scale fabrication

While PSCs have showcased remarkable advancements in small-scale devices, bridging the disparity between laboratory efficiency and large-area devices remains to be a significant challenge. Up to now, the highest efficiency in PSCs has commonly been achieved through a spin coating-based fabrication method. Nevertheless, this particular approach faces significant industrial scalability challenges, primarily stemming from the non-uniform perovskite thin films from center to edge^139^. Additionally, the spin coating process suffers from an exceptionally low material utilization ratio, posing a substantial hindrance to its broader implementation within industrial-scale production endeavors^140^. A multitude of industrially viable alternatives to spin coating methods have been investigated, including doctor blade coating, spray coating, slot-die coating, inkjet printing, and screen printing. These diverse techniques have shown remarkable compatibility with industrial processes, and as a result, substantial advancements have been achieved in the upscaling of PSCs.

#### Different fabricating techniques

**Fig. 10:**
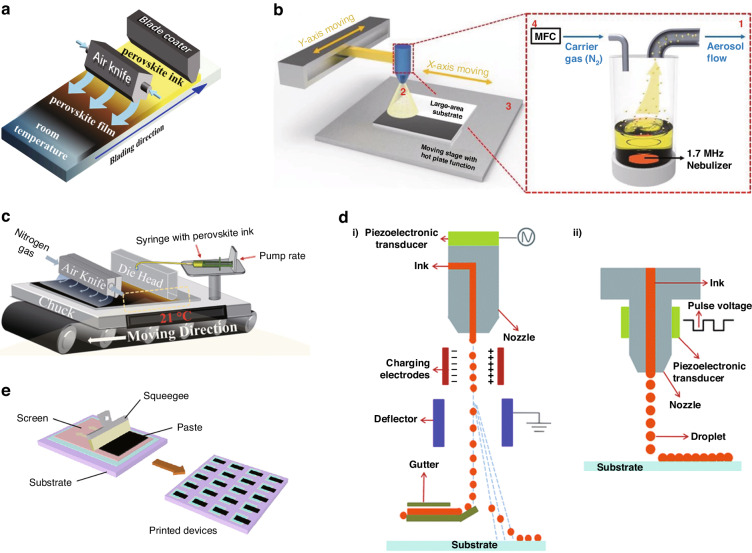
Different fabricating techniques. Schematic illustrations. **a** Air-knife-assisted blade-coating. Reproduced with permission from ref. ^142^ Copyright 2020 Zhengzhou University. **b** Megasonic spray-coating. Reproduced with permission from ref. ^294^ Copyright 2018 Wiley-VCH. **c** Slot die coating. Reproduced with permission from ref. ^150^ Copyright 2021 Wiley-VCH. **d** Inkjet-printing: (i) continuous inkjet printing (CIJ), (**ii**) drop-on-demand (DOD) inkjet printing. Reproduced with permission from ref. ^158^ Copyright Royal Society of Chemistry. **e** Screen printing. Reproduced with permission from ref. ^140^ Copyright 2018 American Chemical Society

In the blade-coating procedure, the initial step involves positioning the substrate onto heated platforms. A specialized blade is then employed to evenly distribute the precursor solution across the substrate’s surface, creating a uniform wet film. The efficacy of this process relies on precisely calibrating the solution’s wettability, thereby enhancing its capacity to coat the substrate comprehensively. As the solvent gradually evaporates, the solute within the solution crystallizes onto the substrate, culminating in the formation of perovskite film. Typically, subsequent annealing is necessary to facilitate crystallization^141^. The quality of the perovskite film is determined by the crucial factors of blading speed, distance between the blade and the substrate, wettability of the substrate, ink viscosity, blading temperature, and crystallization control. In addition to fundamental parameters, the introduction of a nitrogen knife (N_2_-knife) in blade coating expedites the drying of wet films at room temperature (Fig. 10a). It exhibits distinct advantages including room temperature and high-speed coating capabilities. Simultaneously, it generates superior perovskite films with enhanced uniformity and smoothness, even on large-scale substrates, consistently. Precise control of gas blowing conditions—such as gas pressure, nozzle angle, and airflow—is pivotal to this process^142^. The blade coating method offers several benefits over the spinning coating technique for fabricating large-area modules. These advantages include the efficient use of raw materials, the feasibility of preparing in open-air environments, an extended reaction window, and the capability for continuous deposition using Roll to Roll (R2R) and Sheet to Sheet (S2S) processes, underlining its substantial potential for advancement. However, while the extended reaction window in the blade coating method facilitates the growth of perovskite grains, leading to larger grain size, the challenge lies in forming a film free of needle holes during natural drying, primarily due to the slow evaporation of solvents. In recent years, researchers have been diligently working to overcome these challenges. Nowadays, the highest PCE by a blade coating method is 24.31%, which was achieved by a novel pre-seeding approach that involves blending FAPbI_3_ solution with pre-synthesized MAPbI_3_ microcrystals. It can effectively decouple the nucleation and crystallization with the N_2_-assisted blade coating method. Consequently, the initiation of crystallization extends impressively threefold (from 5 s to 20 s), facilitating the creation of homogeneous alloyed-FAMA perovskite films with precise stoichiometric ratios^143^. The largest active area achieved by the blade coating method is 100 cm^2^ ^144^.

Spray coating emerges as a promising technique for upscaling perovskite film fabrication (Fig. 10b). This approach relies on droplets overlapping during contact with the substrate, ensuring uniform film deposition even on curved and non-flat surfaces. The spray coating process unfolds in four distinct stages: droplet generation, droplet transport across the substrate, droplet coalescence into a wet film, and the subsequent drying of this film^145^. Essential spray process parameters, such as substrate temperature, spray head-to-substrate distance, spray head movement speed, and precursor solution dispensing rate, remain consistent across different droplet generation mechanisms. The resultant film morphology is intricately tied to these parameters. Notably, droplet size, ink composition, deposition temperature, drying time, and trajectory significantly influence the formation and overlapping of droplets into thin films^146^. Hence, achieving high-quality perovskite films through spray coating necessitates meticulous control of parameters. Furthermore, a primary drawback of the spray coating method is that newly sprayed droplets tend to dissolve the already-formed film, adversely impacting the quality of the final film coverage. Additionally, the sputtered droplets from this process pose a risk of contaminating the production environment. At present, The highest PCE by a spray coating method is 24.31%, which was achieved by a combination of three key technologies, viz high-performing SAM hole-transport layers, ultrasonic spray coating, and gas-assisted quenching^147^. The largest active area achieved by the spray coating method can reach 112 cm^2^ ^148^.

In slot-die processing, the quality of wet films is determined by the precision of the meniscus formation (Fig. 10c). The distance between the slot-die head and the substrate impacts film thickness, while the balance between pumping rate and coating speed influences coverage, roughness, and uniformity. To expedite wet film drying, slot-die coating facilities incorporate supplementary air-knife or hot-stage features to emulate a quenching effect^149,150^. Furthermore, gas, anti-solvent, and vacuum-assisted quenching techniques prove valuable in achieving large-area films that are uniform, pinhole-free, and consistently homogeneous^151–153^. In contrast to alternative upscaling methods, slot-die coating stands out for its remarkable system compatibility. It seamlessly adapts to both S2S and R2R systems^154^. The slot-die coating method offers distinct advantages over the blade coating technique, particularly in the handling and application of perovskite ink. In this method, the ink is securely stored in a tank, maintaining its viscosity and concentration consistently throughout the coating process. This stability allows for precise control over the film thickness, achieved by adjusting a combination of factors such as the ink’s concentration and viscosity, the distance between the coating head and the substrate, as well as the speeds of both coating and inking and the air-knife pressure. These variables can be meticulously set and programmed in advance. Additionally, a significant benefit of the slot-die method is its non-contact nature; the coating head does not need to touch the base, thereby avoiding potential scratches or damage to the substrate during application. However, to enhance the preparation of superior perovskite films through the slot-die coating method, there is a requirement for more comprehensive studies on fluid dynamics and the design of slot-die apparatus. Up to now, the highest PCE with the slot-die coating method is 23.4%, which is achieved by surface redox engineering for electron-beam evaporated NiO_x_. This strategy not only resolves the issue of local de-wetting in perovskite ink but also significantly boosts the electronic properties at the buried interface by carefully adjusting the surface characteristics of NiO_x_^155^. The largest active area achieved by the slot-die coating method is 300 cm^2^ ^156^.

Inkjet printing stands as a versatile deposition technique, adapted for creating functional layers using consistent molecular or colloidal liquid phase inks. This process functions by expelling ink droplets from a nozzle, allowing for controlled properties and meticulous deposition on a target substrate, ultimately leading to precise fixation. In the realm of inkjet printing, two prevalent techniques have been extensively utilized for generating ink droplets: (i) continuous inkjet printing (CIP) (Fig. 10di) and (ii) drop-on-demand (DOD) inkjet printing (Fig. 10dii)^157,158^. The inject printing method proves highly efficient for producing large-area perovskite films. However, one of the foremost challenges in manufacturing solar cells through inkjet printing technology is developing an ink that not only has the right viscosity and wettability but also maintains long-term stability. Critical to this process is solvent engineering, which involves carefully choosing the ideal solvent or solvent mixtures to achieve the desired ink formulation, taking into account different solubility factors. Additionally, a major hurdle to overcome for the broader adoption of high-resolution inkjet printing is the prevention of nozzle clogging, especially given the need for nozzles with very narrow diameters to achieve high printing accuracy. At present, the highest PCE with the inject printing method is 20.7%, achieved by the oxygen atmosphere tuning during the deposition process and the layer thickness optimization, where the average absorptance of NiO_x_ is just 1%^159^. The largest area with the inject printing method is 804 cm^2^, with a PCE of 17.9%^160^.

In the realm of screen printing, a squeegee exerts pressure to transfer paste onto a substrate through openings in a meticulously patterned mesh screen, crafted from either fiber or steel mesh (Fig. 10e). The final thickness of the printed films hinges on factors such as mesh size, screen thickness, and the paste material ratio. Screen printing stands out as a fast and flexible method for transferring diverse patterns onto a variety of substrates. It is highly compatible with different functional layers, offering considerable flexibility in pattern design and scalability. This method holds great potential for a wide range of applications. Up to now, the highest PCE with inject printing method is 20.52%^161^. The largest area with the screen printing method is 198 cm^2^ ^162^.

The description provided suggests that various fabrication techniques lead to differing gaps between the lab PCE and PSM. In the next section, we focus on elucidating the considerations surrounding the scaling-up factor and the associated cost factor pertaining to PSM.

#### Some factors in PSM

The increase in the perovskite film’s surface area corresponds to a higher defect density and diminished film uniformity, ultimately resulting in a decline in the photovoltaic performance of PSM. When contrasting the photovoltaic performance of films with varying sizes, it is difficult to make a fair and consistent comparison of their performance disparities. Hence, formula (4) served as the means to ascertain the scaling-up factor (*f*_scaling-up_), a pivotal determinant in gauging upscaling losses^163^. The formula is presented below:

4 $$f=\left[\left(1-\frac{{\eta }_{{module}}}{{\eta }_{{cell}}}\right)\times 100 \% \right]/\left[\log \frac{{A}_{{module}}}{{A}_{{cell}}}\right]$$

where *η* is the PCE, and A is the active area of the cell or module. The lower the value of *f*_scaling-up_, the less amplification loss occurs from PSCs to PSM, making it increasingly favorable for the industrial advancement of PSM technology. The highest lab PCE of PSCs and the PCE of the biggest area of PSM up to now are shown in Table 1.

**Table 1:** Reported highest lab PCE of PSCs and the PCE of the biggest area of PSM

		Active area (cm^2^)	PCE (%)	*f*_scaling-up_ (%)	Ref.
Blade Coating	Cell	0.04	24.31	24.22	^143^
	Module	100	4.3		^144^
Spray Coating	Cell	0.025	20.8	9.19	^147^
	Module	112	13.82		^148^
Slot-die Coating	Cell	0.1	23.4	14.99	^155^
	Module	300	11.2		^156^
Inject Printing	Cell	0.105	20.7	3.48	^159^
	Module	804	17.9		^160^
Screen Printing	Cell	0.05	20.52	18.86	^161^
	Module	198	6.6		^162^
Evaporation	Cell	0.1	24.42	19.72	^281^
	Module	82.6	10.37		^284^

From Table 1 we can find that the value of *f*_scaling-up_ for inject printing is the lowest, which indicates the amplification loss can be effectively minimized by inject printing method. In the course of amplifying devices, a loss of efficiency is inevitable. Consequently, the pressing challenge remains to develop a PSM that is both compatible with large areas and capable of achieving high efficiency. By contrast, the PCE of silicon heterojunction solar cells reached 26.81%, with an area of 274.4 cm^2^ ^164^. While the PCE of PSCs has rapidly approached that of silicon solar cells, a significant disparity in the context of large-area modules still exists. Hence, there is an ongoing imperative to enhance both the functional layer and the preparation process of PSMs so as to narrow down this existing gap.

Table 2 shows the data of PSCs operated at MPPT for more than 2000 h, from which we can find that the maximum running time of the existing PSCs is 9000 h^115^. The durability of silicon solar cells significantly surpasses that of PSCs in terms of operational lifespan. What’s more, to address the substantial variations in ambient temperatures during different operational conditions (temperature, humidity, etc.), the establishment of a standardized metric becomes imperative for assessing the long-term operational stability of the device. Such a metric would serve as a pivotal reference point for its prospective industrial applications.

**Table 2:** Statistics for PSCs data as the PSCs operated at MPPT for more than 2000 h

Device structure	Operation temperature	Operation time	Ref.
ITO/SnO_2_/FA_1-x-y_MA_x_Cs_y_PbI_3-z_Br_z_/Spiro-OMeTAD/Au	RT	*T*_95_ = 2000 h	^285^
FTO/TiO_2_/Al_2_O_3_/CsPbI_3_/CuSCN/(Cr/Au)	110 °C	*T*_80_ = 2100 h	^286^
ITO/MeO-2PACZ/Rb_0.05_Cs_0.05_MA_0.05_FA0_.85_Pb(I_0.95_Br_0.05_)_3_/LiF/C60/BCP/Ag	55 °C	*T*_87_ = 2428 h	^287^
FTO/SnO_2_/Cs_5_(MA_0.10_FA_0.90_)_95_Pb(I_0.90_Br_0.10_)_3_/spiro-OMeTAD/Au	60 °C	*T*_99_ = 2000 h	^288^
ITO/SnO_2_/Cs_0.1_FA_0.9_PbI_3_/spiro-OMeTAD/Au	45 °C	T_91_ = 3190	^289^
ITO/SnO_2_/CsFAMAPb(I_1−*x*_Br_*x*_)_3_/spiro-OMeTAD/(Au/Ag)	70 °C	*T*_95_ = 3265 h	^290^
ITO/SnO_2_/FAPb(I_*x*_Br_1−*x*_)_3_/spiro-OMeTAD/C	60 °C	*T*_90_ = 4390 h	^291^
FTO/NiO_*x*_/Me-4PACz//FA_0.95_Cs_0.05_PbI_3_/PEAI/C_60_/SnO_2_/Ag	40 °C	*T*_100_ = 3500 h	^292^
FTO/TiO_2_/ZrO_2_/C	55 °C	*T*_95_ = 9000 h	^115^

The cost of preparing PSM plays a crucial role in its path to industrialization. While the preparation of PSM is straightforward and comparatively less expensive than traditional silicon solar cells, continually driving down costs remains paramount for its successful industrial advancement. However, the recycling of PSCs is still necessary for sustainable development. The dismantled PSCs are classified into two groups: non-hazardous materials (including FTO glass and metal electrodes) and hazardous materials (including lead-containing compounds). The conductive substrates, obtained through thermal, mechanical, or chemical separation methods, can be reclaimed following cleansing and restoration procedures. Incorporating these reclaimed substrates into PSC production yields noteworthy reductions in material expenses, thereby enhancing economic gains. Furthermore, recycling the top electrodes in PSMs—materials like Au, Ag, Cu, Al, and Ni—offers substantial cost savings in manufacturing when contrasted with acquiring new precious metal resources. On the other hand, recycling lead-containing materials not only slashes costs but also significantly mitigates the environmental impact of lead, a subject that will be expounded upon in the following chapter. Beyond recycling in the end product, minimizing costs at the source represents a more immediate and efficacious approach. Han’s group has significantly slashed the preparation expenses for PSM by forsaking the conventional costly HTL and substituting the expensive gold electrode with an affordable carbon electrode. This unique three-layer mesoporous membrane structure exhibits substantial potential for industrial development as a device architecture^165^.

Generally, achieving industrial development for PSCs hinges on these crucial factors: minimizing scaling-up factors, extending the device’s operational lifespan, and reducing its preparation costs. In the industrialization of large-scale perovskite devices, it is crucial to factor in both cost-efficiency and environmental considerations during the manufacturing process. Achieving industrial-scale production necessitates the development of a streamlined and simpler preparation process. This approach should enable the efficient and cost-effective fabrication of high-quality perovskite devices.

## Multi-scenario applications of perovskite solar cells

**Fig. 11:**
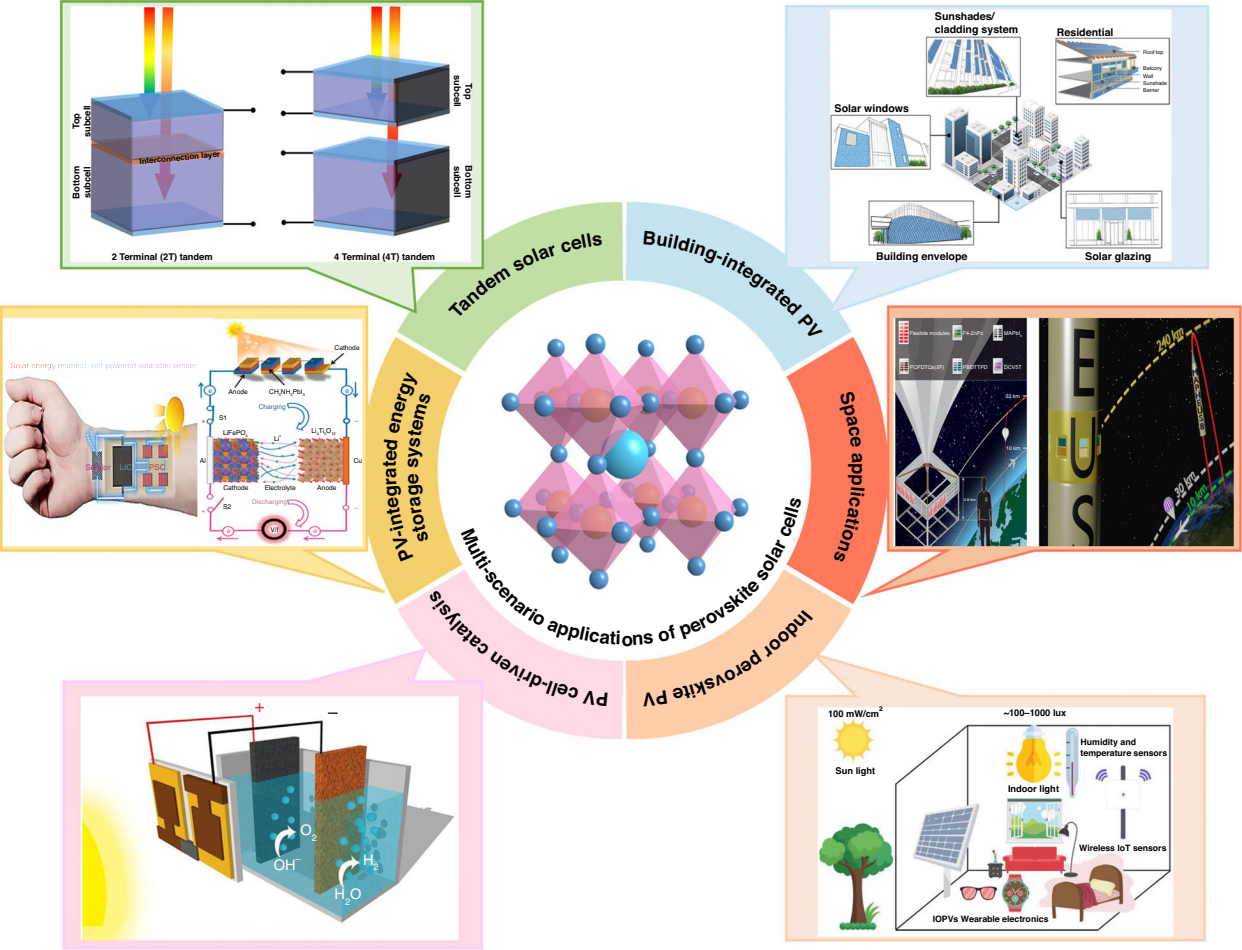
Schematic overview of the application covered in this section. Reproduced with permission from ref. ^176,177,179,187,295–297^ Copyright 2023 Springer Nature, 2022 Wiley-VCH, 2019 Elsevier Ltd, 2021 Wiley, 2014 American Association for Advancement of Science and 2021 Wiley-VCH

**Fig. 12:**
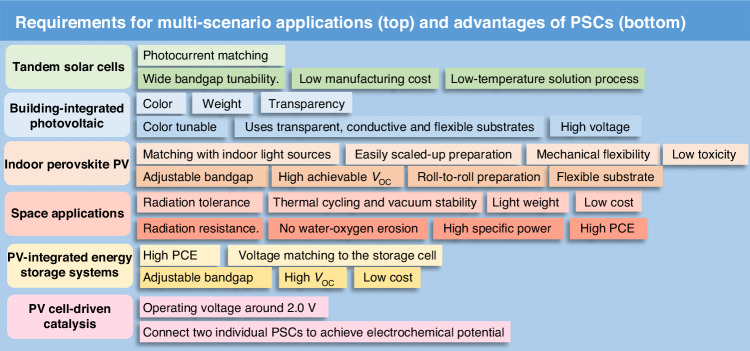
A summary of the requirements for multi-scenario applications (light color)

In recent years, the efficiency of PSCs has improved by leaps and bounds to a similar level as silicon cells. This has led to a consensus that PSCs are the most promising next-generation photovoltaic for industrialization. Moreover, PSCs are available in a wide range of fabrication techniques and device structures, which can meet the application requirements of multiple scenarios. Therefore, researchers have made a variety of attempts on the real-life applications of PSCs, including tandem solar cells^166–168^, building-integrated PV^169–171^, indoor photovoltaics^172,173^, space applications^174–176^, PSC-integrated energy storage systems^177–179^, and PSC-driven catalytic systems^180–184^ (Figs. 11 and 12). In this section, the requirements in PSC applications and the advantages of PSCs were discussed and the newest achievements that have been made were presented. Finally, we are looking ahead to the challenges and prospects of the promising PV-integrated new technologies.

### Building-integrated photovoltaics

**Fig. 13:**
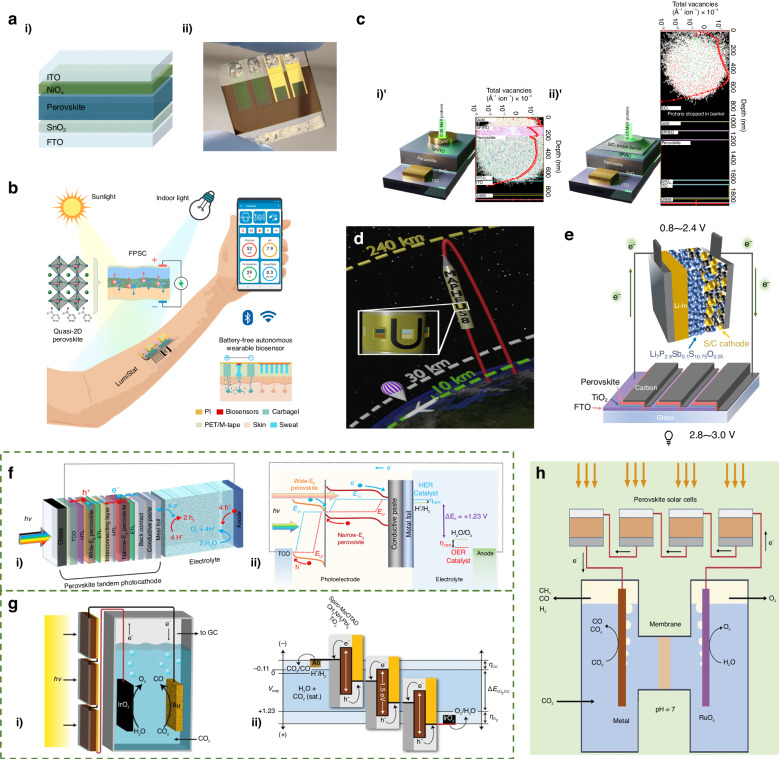
The progress in the application of PSCs. **a** (i) Configuration and (ii) photograph of the semi-transparent PSC with n-i-p structure. Reproduced with permission from ref. ^186^ Copyright 2022 Wiley-VCH. **b** Illustration of the energy-autonomous wearable device that is powered under both outdoor and indoor illumination through a quasi-2D FPSC. Reproduced with permission from ref. ^189^ Copyright 2023 Springer Nature. **c** Proton straggling in an n-i-p device without (i) and with (ii) a 1-μm-thick SiO_x_ proton barrier. Reproduced with permission from ref. ^192^ Copyright 2023 Springer Nature. **d** Schematic Overview of the rocket flight with PSCs. Reproduced with permission from ref. ^193^ Copyright 2020 Elsevier Ltd. **e** Diagram illustrating the design of an all-solid-state Li−S battery with photo-rechargeable capabilities. Reproduced with permission from ref. ^197^ Copyright 2022 Elsevier Ltd. **f** Schematic illustration of (i) the device structure of a double-junction all-perovskite tandem photocathode and (ii) the energy band diagram for water splitting. Reproduced with permission from ref. ^200^ Copyright 2023 American Chemical Society. **g** (i) Schematic of the device combining photovoltaics with an electrochemical cell and (ii) the generalized energy diagram for converting CO_2_ into CO with three PSCs. Reproduced with permission from ref. ^202^ Copyright 2015 Springer Nature. **h** Diagram depicting the light-driven electrochemical apparatus designed for the conversion of CO_2_ to CO. Reproduced with permission from ref. ^204^ Copyright 2020 Elsevier Ltd

The adjustable band gap of perovskite materials can meet the spectrum of different light sources; thus, PSCs can be applied outdoors, indoors, and even in space. The top and side facades of buildings have a long time and high intensity of sunlight, which is a good place to install PV devices. Building-integrated PV can turn the building structure into a generator to provide electricity for the building. However, the locations of windows and facades put additional requirements on PV devices, such as transparency, weight, shape, and color. This results in rigid Si solar cells not being suitable for the task, and PSCs, with the advantages of color tunability, substrate transparency and flexibility, and adjustable transparency, are the best candidates for building-integrated PV. The perovskite devices commonly used for building-integrated PV are semi-transparent and colored for partial light transmission. For semi-transparent PSCs, there exists a balance between PCE and average visible transmittance (AVT). Therefore, the PCE of semi-transparent PSCs is only close to 15% (AVT = 20%) which is much lower than non-transparent PSCs^185^. To improve the PCE, the use of transparent HTL or electrodes to enhance the light utilization of the device, and the improvement of the quality of perovskite to strengthen light absorption are effective strategies. Jun Hong Noh’s group reported a structure of perovskite/p-type oxide (NiO_x_)/n-type oxide (ITO) (Fig. 13a)^186^. The p-type NiO_x_ nanoparticle layer covered with perovskite served as a transparent HTL and buffer layer to avoid sputtering damage to perovskite during the deposition of transparent conductive oxides. Meanwhile, ITO was used as the top transparent electrode to maximize the light utilization capability of PSCs. Benefiting from this, the semi-transparent device obtained a PCE of 19.5%, which is even higher than the PCE of 19.2% for the non-transparent device. In addition, a semi-transparent device with AVT = 30.03% can be obtained by reducing the concentration of the perovskite precursor solution. The above structure improves the light utilization ability while ensuring the quality of perovskite, which provides a foundation for the industrial application of semi-transparent PSCs. For colorful PSCs, the colors presented by transmission and reflection can be adjusted by changing the perovskite band gap and transparent electrode thickness and by using optical nanostructures, optical microcavities, and photonic crystals. However, realizing colorful PSCs reduces the light-harvesting ability of the device and sacrifices the thickness of the perovskite layer which weakens the light-absorbing ability of perovskite. Thus, the PCE of colorful PSCs remains a barrier to industrialization. Increasing PCE with a guaranteed 25% AVT can only offset the effect of low *J*_SC_ by increasing *V*_OC_ and FF.

To summarize, researchers have made efforts to address the key challenges in building integrated PV, but this has brought about other problems at the same time. Currently, the cost of PSCs for building-integrated PV outweighs the benefits, it will take a long time to find the optimal answer to the balance between PCE and light transmittance/color.

### Indoor photovoltaics

The rapidly growing Internet of Things (IoT) requires a continuous electrical power supply, which is driving the indoor application of flexible perovskite photovoltaics that meet its requirements. As PV devices are located indoors, they need to fulfill the following requirements: matching with indoor light sources, matching with IoT voltages, low toxicity, and mechanical flexibility. Bandgap tunable and high-voltage flexible perovskite devices are suitable for these requirements. Due to fewer photons received by the device, the indoor PV produces less output power^187^. At the present stage, the PCE of Cs_0.17_FA_0.83_PbI_x_Br_3-x_ indoor PV has already achieved 36.36%^188^. Reducing the defect density of perovskite with composite and interfacial engineering to suppress the leakage current and optimizing the perovskite composition to match the visible emission spectrum of the indoor light source can achieve a significant increase in PCE. Perovskite indoor photovoltaics have already met the initial requirements in efficiency, but it is the stability that is critical for sustained power generation. Compared to outdoors, light and heat are mild indoors. Therefore, the degradation mechanism of indoor PV is different from that of outdoor. It is mainly the production of a few photoelectrons that allows partial filling of the trap state thus accelerating the long-term degradation^187^. The strategies such as cation and anion combination engineering, additive engineering, anti-solvent engineering, and defect management effectively extend the lifetime of perovskite indoor PV. As shown in Fig. 13b, Min et al.^189^ have attempted to use a flexible quasi-2D perovskite solar cell module for self-powering wearable biosensors that provide continuous and non-invasive metabolic monitoring. The flexible device provided sufficient power under both outdoor and indoor conditions (PCE over 31% under indoor light illumination) to maintain the biosensor working continuously for 12 h. This work confirms the feasibility of integrating PSCs with multiple indoor devices.

Despite the impressive progress that has been gained in indoor photovoltaics, there are still problems in several areas. First, the inconsistency between indoor and outdoor light sources and environments has brought about changes in the concentration of charge carriers and recombination dynamics^190^. Under indoor light source irradiation, the carrier concentration is much lower than that under solar illumination. As a result, bimolecular recombination is naturally mitigated and trap-induced recombination is amplified. The corresponding device performance is affected by the shunt resistance (*R*_SH_), which modulates both FF and *V*_OC_. Under solar radiation, the effect of the trap state in the films is hidden by the high charge density and bimolecular recombination dominates. The corresponding device performance is mainly affected by the series resistance (*R*_S_). When *R*_S_ increases, FF and *J*_SC_ decrease. Second, relevant measurements have to be performed to simulate real indoor conditions and to establish a common standard. In addition, the toxic effects of the perovskite devices are amplified indoors. If the above problems are solved, the development of perovskite indoor PV will even surpass that of outdoor PV.

### Space application

Perovskite photovoltaics with radiation tolerance and defect tolerance have attracted attention for space applications as well. The water-free, oxygen-free, and shade-free space environment seems to mitigate the degradation of PSCs. However, the space environment brings other challenges such as particle radiation, high UV radiation, thermal cycling, and vacuum stability. In addition, space applications involve the process of being carried by space equipment, so PV devices are required to be lightweight, low-cost, and mechanically flexible. Although Si and III-V multi-junction compound solar cells (CdTe, GaAs, CIGS) are currently used for most of the space PVs, their high preparation cost, high weight, and rigid structure all lead to low energy efficiency. Therefore, suitable alternatives for Si and III-V multi-junction compound solar cells need to be found. The flexible PSCs with low cost and high specific power (i.e., power-to-weight ratio) are an ideal choice. Reb et al.^191^ mounted standard PSCs based on mesoporous TiO_2_ and SnO_2_ on a suborbital rocket to observe device performance. The devices showed satisfactory performance (power density over 14 mW·cm^−2^) while the rocket reached the highest point of 239 kilometers for 6 minutes with temperatures ranging from 30 °C to 60 °C. This time of observation, though short, proved that PSCs have a promising future for space applications. The most significant problem faced by perovskites in space is radiation. When energetically charged particles interact with a material, they transfer and deposit their momentum and energy into the material. The irradiated material produces defects in the internal molecular structure due to ionization and atomic displacement, and the encapsulation glass also turns black. This leads to poor performance and even damage to semiconductor devices. Recently, Kirmani et al. evaporated a 1 μm thick silicon oxide layer on the top of devices that acts as a barrier layer (Fig. 13c)^192^. It protected the device from damage blocking 0.05 MeV protons at an injection of 10^15 ^cm^−2^. The device was even exposed to α-radiation and atomic oxygen without degradation of PCE. Meanwhile, this led to an increase in device lifetime up to 20 and 30 times in low- and high-Earth orbits, respectively. This barrier technology is a critical step for PSCs toward space applications. On August 29, 2021, the on-board PSCs were flown in low Earth orbit (LEO) outside the International Space Station (ISS) for six months during the 15th Materials ISS Experiment (MISSE-15) (Fig. 13d)^193^. This was the first long-term flight of PSCs in LEO, further confirming that PSCs can survive in the space environment. However, the high vacuum and high-temperature characteristics of space make the stability of perovskite films challenging. Under extreme conditions, the MA-based perovskite film degrades into gaseous products, and a large number of holes are created in the perovskite film^194^. With the help of the Cs shrinking lattice, CsFAMA-based mixed cationic perovskite films show promising stability compared to MA-based perovskite^195^. Comparison and optimization of multiple perovskite materials may be an effective way to enhance stability.

Space applications of PSCs have been initially developed, but some challenges have yet to be overcome. For example, the standards for evaluating solar cells in space applications are the AIAA-S111 eligibility standard based on Si and III-V materials^196^, which are not fully suitable for perovskite; the damage mechanisms of PSCs by energetic particles and radiation are not yet clear; simulating realistic outer-space conditions is difficult; and how to design the appropriate devices and encapsulations. The PSC with unique advantages has given hope for the implementation of photovoltaics in space, which is possibly the next generation of space solar cells.

### PV-integrated energy storage and fuel conversion systems

The periodic variations in the intensity of solar irradiation make it impossible for solar cells to consistently generate electricity at maximum power. In addition, solar cells need to consume power immediately from the conversion of light to electricity. In order to address the immediate and intermittent disadvantages of PV power generation, storing electrical energy in energy storage devices or converting it into easily storable clean energy with high energy density is the ideal strategy. The former is generally integrated with rechargeable batteries having high energy and power density, such as supercapacitors and lithium batteries, into PV-integrated energy storage systems. The latter commonly utilizes electrocatalytic reactions to prepare fuel compounds, for instance, the decomposition of water to prepare hydrogen and the reduction of CO_2_ to hydrocarbons.

The first thing needed in integrating PV with them is to meet their operating voltages, i.e., voltage matching. In the past, lithium batteries have been attempted to be integrated with Si-based and dye-sensitized solar cells (DSSCs). However, the low voltage of their single-junction cells (less than 0.8 V) requires a tandem of several cells for integration to work, which brings cost and size issues. With the advantages of *V*_OC_ over 1 V and low cost, PSCs are certainly more suitable than Si cells and DSSCs. Recently, Li et al.^197^ demonstrated an indoor energy harvesting and storage system employing an all-solid-state photo-rechargeable battery. It comprises an all-inorganic CsPbI_2_Br PSM and an all-solid-state lithium-sulfur battery (Fig. 13e). The energy conversion and storage unit exhibited an excellent overall energy conversion and storage efficiency of 11.2% and a high electrochemical storage ability of 1585.3 mAh/g under LED illumination. In addition, the device exhibited good safety and stability after a 200-h photo-charging and constant-current discharge cycle. If the stable operation time can be further increased, the photo-rechargeable battery system can be applied in more fields. Therefore, trying more efficient and stable PSCs and more severe tests, such as moisture, thermal, long-term operation, and charge/discharge cycle tests, are helpful for the photo-rechargeable battery system to move toward practical applications.

The device consisting of a PSC and a supercapacitor is called a photo-supercapacitor. Supercapacitors have the advantages of ultra-long cycle stability, fast charge/discharge, and high-power density, but integration with it requires high operation voltages. Therefore, enlarging the active area of PSCs or tandem connecting individual photo-supercapacitors is a good solution. Liu et al.^198^ integrated a PSC with a supercapacitor based on a normal carbon electrode and demonstrated a tandem system based on four individual devices (with an active area of 7.5 cm^2^). The system can quickly reach a stable output voltage of about 3.8 V and drive a light-emitting diode under 1.0 sun. Although it only sustained for a few minutes due to the degradation of PSCs, this also showed that PSC-based photo-supercapacitors have potential for applications in self-powered electronic devices and portable power sources.

The PSC-driven catalytic systems need the PV electrolyzer to provide a voltage greater than the reaction overpotential. Generally, the operating voltage for water-splitting should exceed *V* = 1.23 + *φ*_HER_ + *φ*_OER_^199^. Similarly, for CO_2_ reduction to CO, the operating voltage for CO_2_ reduction should exceed *V* = 1.34 + *η*_Cathode_ + *η*_Anode_^199^. The *V*_OC_ of PSCs is generally more than 1 V so it is anticipated that only 2 PSCs in tandem can provide the required operating voltage. The fabrication of a monolithic integrated all-perovskite stacked photocathode was reported by Song et al. (Fig. 13f)^200^. The all-perovskite tandem photocathode connected with an iridium oxide anode provided high photovoltaic voltages over 2 V at zero applied bias under AM1.5 G 1 sun illumination. Hence, the PSC-driven catalytic systems yielded a solar-to-hydrogen (STH) conversion efficiency of 15%. Moreover, it can operate continuously in water for more than 120 h under simulated 1 sun illumination with less than 5% efficiency loss. At present, PSCs-driven catalytic systems still have relatively low STHs and short lifetimes, which makes them cost-inefficient. Therefore, it is crucial to improve the STH and stability. The STH is mainly affected by PSC photovoltaic conversion efficiency and catalytic reaction rate. The supply of efficient and stable photovoltaic devices and bifunctional catalysts holds the promise of bringing efficient, durable, and cost-effective photo-hydrogen production technology. Recently, Michael Wong & Aditya D. Mohite achieved a record STH efficiency of 20.8% and 102 h of continuous operation under AM1.5 G illumination using a monolithic perovskite/Si tandem as a photovoltaic anode and IrO_x_-coated conductive adhesive barriers (CABs) with Pt foils as cathodes^201^. The high STH was attributed to the high PCE of about 30% of PSC and the stable catalysis for reduction and oxidation reactions by CAB/catalyst. In addition, the CAB played the role of a protective barrier for the photocathode and anode, which not only remains unaffected in the photovoltaic performance of the PSC but also provides a high device lifetime while minimizing the cost. A techno-economic analysis showed that if a sufficiently long lifetime can be achieved, it is expected to keep the levelized cost of hydrogen below $1/kg^200^. This work provides a pathway towards inexpensive solar hydrogen production.

Converting CO_2_ to fuel using solar energy may assist in CO_2_ reduction and even achieve CO_2_-CO-CO_2_ recycling, thus weakening the greenhouse effect. However, the higher overpotential and lower solar-to-CO (STC) efficiency of the CO_2_ reduction reaction compared to water-splitting makes the PV-driven reaction more challenging. In order to meet the high voltage (>2 V) requirement, an enough number of single-junction PSCs are generally connected in tandem. Schreier et al.^202^ employed 3 tandem-PSCs in combination with gold oxide electrodes and IrO_2_ anodes to obtain an STC > 6.5% (Fig. 13g). This work created a good demonstration for PSCs-driven CO_2_ reduction, but there was still a room for improvement in STC. Compact electrochemical cell design, large-area electrodes and perovskite films, suitable pH environment, and electrode selection were found to reduce overpotential and improve product selectivity. Huan et al.^203^ combined a stack of PSCs in series and parallel with a continuous flow electrochemical cell to minimize mass transfer limitations of CO_2_ and manage resistance losses. The total efficiency of solar energy conversion to hydrocarbons was 2.3%. Similarly, Esiner et al.^204^ demonstrated unassisted light-driven electrochemical CO_2_ reduction to CO and CH_4_ with 4 series-connected PSCs (Fig. 13h). They achieved 8.9% STC and 2% solar-to-CH_4_ conversion efficiency after 7–10 h. Including H_2_ production, the total solar-to-fuel conversion efficiencies for 10 h were 8.3%–9.0%.

Taken together, these results indicate that PSCs-driven CO_2_ reduction reaction is still cost-inefficient as well. Therefore, electrochemical cells and efficient catalysts need to be designed based on the characteristics of CO_2_ reduction reactions to improve the conversion efficiency. Moreover, the pursuit of affordable, high-performance substitutes for expensive metal electrodes could further drive cost reduction.

## Sustainability issues of perovskite solar cells

PSCs represent a significant breakthrough in the field of solar technology, with notable potential for sustainable development. These novel solar cells offer high energy conversion efficiency, relatively low manufacturing costs, and a wide range of potential applications. To achieve their sustainable development, a series of key measures must be taken. Besides the demand that research and development should be more stable, long-lasting perovskite materials to extend the lifespan of the cells and reduce resource waste, continuously improving the production process of PSCs and minimizing the environmental impacts is of the utmost importance. In the production process of PSCs, pollution arises from two primary sources: lead and hazardous solvents.

### Environmental issues of lead

#### The adverse effects of lead toxicity

Since the photoelectric properties and thermodynamic and environmental stability of non-lead perovskites cannot be compared with lead halide perovskites at present, the heavy metal lead in perovskites is still irreplaceable^205–209^. However, perovskite will be degraded to lead iodide (PbI_2_) by light, heat, and humidity in long-term operation. Hydrogen bonding of lead iodide with water makes it soluble in water, and longer rainfall times (>10 min) can even completely dissolve the perovskite film when the device is damaged^210^. To assess the extent of the environmental impact of lead in PSCs, the lead content of the device was first estimated. A typical flat-plate device has a perovskite layer thickness of about 500 nm, then the area densities of lead corresponding to MAPbI_3_, FAPbI_3_, and CsPbI_3_ in it are about 0.066 mg/cm^2^, 0.067 mg/cm^2^, and 0.077 mg/cm^2^ ^211,212^. Comparing with the lead content of solder required for Si solar cells that have been industrialized so far (0.61 mg/cm^2^), the lead content of PSCs is an order of magnitude smaller. The PSCs also meet the EU legislation requirement that “homogeneous materials” contain no more than 0.1% lead by weight^213^. If the lead in the PSC module with MAPbI_3_ component is dispersed into the same area of soil below, the lead content in the soil will increase by 4.0 mg/kg, which is far below the upper limit of 250 mg/kg for agricultural soil in China^211,214^.

**Fig. 14:**
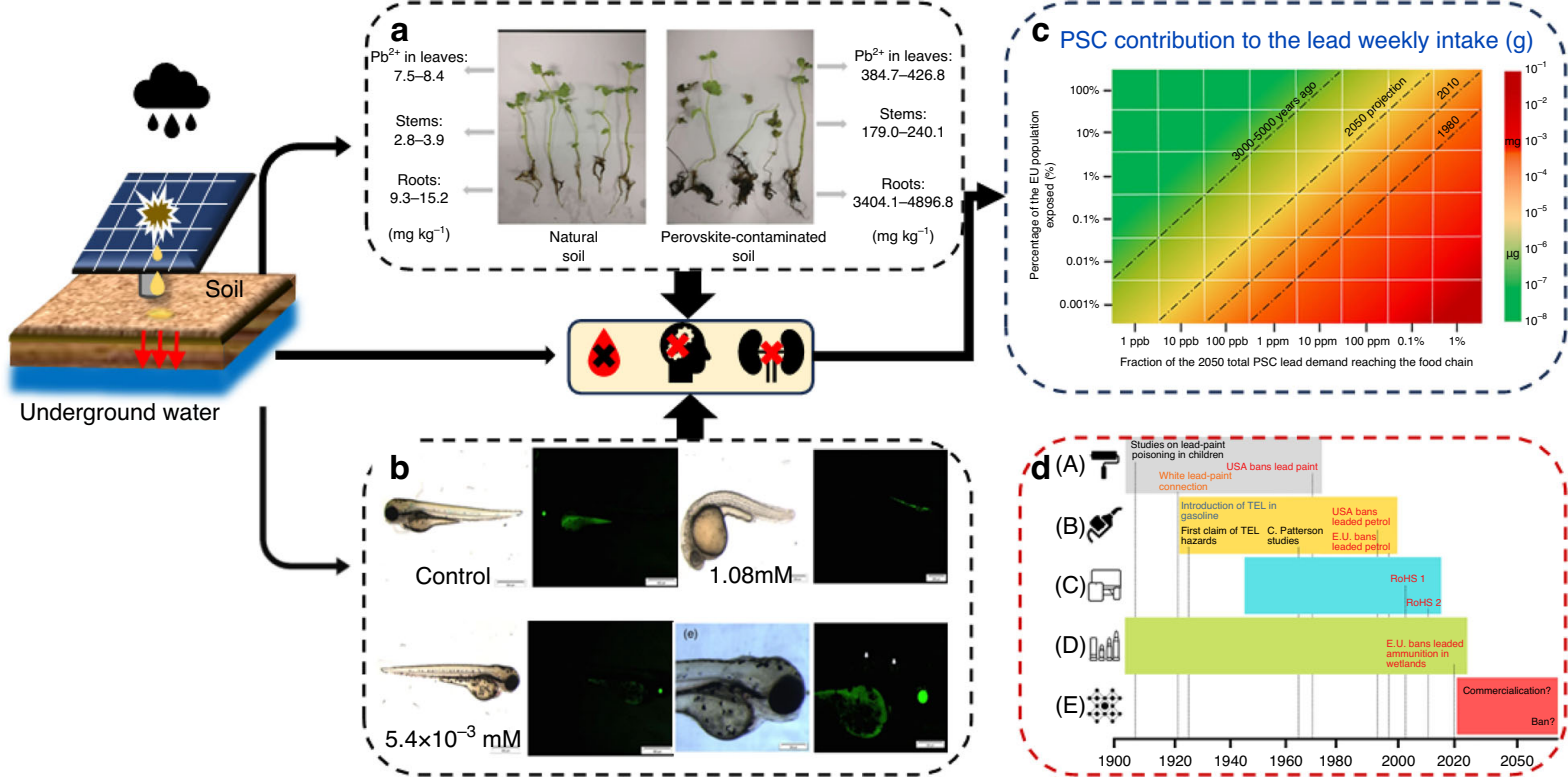
Lead damage from perovskite solar cells. **a** Comparison of mint plants cultivated in two different soil conditions: control soil (left) and soil contaminated with 250 mg kg^−1^ Pb^2+^ perovskite (right). The range lead content in the leaves, stem, and root is indicated alongside each image. Reproduced with permission from ref. ^211^ Copyright 2020 Springer Nature. **b** Stereoscopic (left) and fluorescent (right) images showing Tg (hsp70l: GFP) embryos treated with different concentrations of PbI_2_. Reproduced with permission from ref. ^215^ Copyright 2016 Springer Nature. **c** Heat map displaying various scenarios of lead exposure to analyze potential solar plant failures. It considers the percentage of lead entering the food chain (indicating the number of panels failing) and the percentage of the European Union (EU) population exposed to this lead contamination (reflecting the extent of environmental lead spread). **d** Evolutionary timeline of the regulation of Pb-containing products. Reproduced with permission from ref. ^223^ Copyright 2022 Elsevier Ltd

The above results seem to make it easy to assume that the lead content in PSCs is low and will not cause serious consequences. However further considering the bioavailability, the problem becomes critical. Li et al. measured the uptake of Pb by plants by growing them in Pb-contaminated soil^211^. They found that the bioavailability of Pb to plants was enhanced by a factor of 10 due to the effect of organic cations on soil pH, i.e. increasing the effective Pb concentration in soil by only 10% increased the Pb content in plants by more than 100%. In particular, at 250 mg/kg of soil Pb, plants already showed blackening and decay (Fig. 14a). To further understand the effects of lead toxicity on organisms, Babayigit et al. performed a Zebrafish embryo acute toxicity testing (ZFET) protocol which was with 85% genetic similarity to humans^215^. Figure 14b shows stereoscopic and fluorescent pictures of transgenic embryos exposed to the chemical. For normal control embryos, fluorescent signals can be found in the yolk and lens regions. When exposed to PbI_2_, the combined effect of heavy metals and their resulting pH reduction can be observed. Embryos exhibit multiple defect types, such as increased fluorescence in the trunk curvature region and dim fluorescence in the head and neck, corresponding to dorsal curvature and cerebrovascular defects, respectively. Zeng et al. directly accounted for the toxic effects of Pb on children from an e-waste disposal area^216^. By examining blood and urinary lead concentrations and microbiota and metabolites in fecal samples of children, they came to the conclusion that high blood and urinary lead levels caused by the Pb exposure group were positively correlated with a significant reduction in gut microbial diversity and significant changes in metabolites. In addition to this, Pb^2+^, due to their similarity to biologically essential ions (Ca^2+^/Fe^2+^/Zn^2+^), occupy their binding sites, which in turn affects normal physiological processes^217,218^. Excessive levels of lead in the body affect the work of the hematopoietic system, inhibit the development of the nervous system, and damage the digestive system^219–221^. It has a significant impact on children in particular, not only hindering intellectual development but also leading to tooth decay and learning behavioral abnormalities^216^. The order of toxicity of different types of lead sources is Pb^2+^>PSC>PbI_2_ = PbO^222^.

As noted above, the amount of Pb leakage from just one PSC device will not cause significant environmentally undesirable results. However, after a large-scale installation of PSC components, the total amount of Pb leakage will increase by orders of magnitude with the area of use. At the same time, fixed module installation locations (soil, roof, water) can result in the same place being subjected to Pb leakage for a long period of time, causing regional environmental degradation. In addition, contaminated soil and water can easily exceed the lead content of organisms, and they can enter the food cycle to further accumulate in the human body. Lead weekly intake (LWI) levels were estimated for different percentages of lead reaching the food chain (i.e., number of faulty panels) and for different percentages of the EU population exposed to lead (i.e., the extent to which the leaked lead has diffused into the environment), as shown in Fig. 14c^223^. Due to the fixed installation nature of PSCs components at specific locations, lead leaks should be regional in nature. Then even if the percentage of lead that can reach the food chain is 10^−10^, it will still exceed the FAO limit of 0.025 mg/kg. Therefore, it is important to be concerned about the heavy metal lead in calcite, stop the lead leakage, and recycle it efficiently to reduce our exposure to lead. Many lead-containing products (e.g., paint, gasoline, ammunition) were used on a large scale without concern for lead, and were eventually banned with serious consequences (Fig. 14d). Hence, researchers have been aware of the problems posed by lead in PSCs and have adopted a number of strategies to inhibit lead leakage, adsorb lead that has leaked, and recycle and reuse lead from end-of-life devices.

#### Stopping lead leakage into the environment: encapsulation technology

**Fig. 15:**
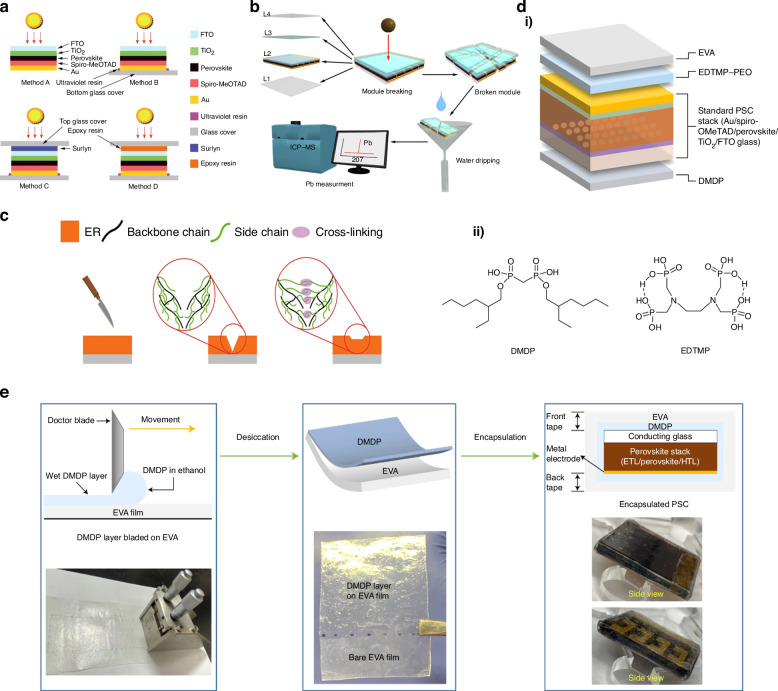
Physical encapsulation to stop lead leakage. **a** Illustration depicting various encapsulation techniques. and **b** Illustration outlining the experimental process for quantifying the amount of toxic lead (Pb) leakage from a module subjected to a standard hail impact test (FM 44787). **c** The self-healing mechanism of the ER encapsulant. Reproduced with permission from ref. ^225^ Copyright 2019 Springer Nature. **d** Diagram depicting Pb-absorbing materials, with DMDP film on the front (glass) side and EDTMP-PEO film on the rear (metal electrode) side. Reproduced with permission from ref. ^226^ Copyright 2020 Springer Nature. **e** The schematic representation of applying DMDP using the doctor-blading technique onto an EVA film (top). The encapsulation of the fabricated PSC, with DMDP-laminated EVA tapes applied to both sides (top). Reproduced with permission from ref. ^10^ Copyright 2021 Springer Nature

Physical encapsulation is a common approach to improve the operational stability of PSCs, avoiding environmental erosion and enhancing the module’s impact resistance^9^. However, the general physical encapsulation only adopts a five-layer stacked structure of glass/EVA (surlyn)/PSC/EVA/glass, which can still be severely damaged under extreme conditions such as hailstorms and fires, leading to lead leakage^224^. Jiang et al.^225^ first introduced epoxy resin-based polymer (ER) as encapsulation layers and compared them with common physical encapsulation methods (Fig. 15a). They found that ER not only dramatically increased the mechanical strength of modules, but also allowed for self-healing at high temperatures (Fig. 15b). The encapsulated PSM was damaged by a simulated standard hail impact test (FM 44787), followed by an acid rain simulation test. The test results showed that the lead leakage rate of the target group was reduced by a factor of 375 (Fig. 15c). Therefore, suitable physical encapsulation techniques can prevent the transfer of lead to the environment. In addition, they noted that encapsulation method, weather conditions, and response time were the main factors affecting lead leakage. Lead leakage in cold weather has rarely been studied, but at this time devices are more likely to be damaged and remain in contact with snow and water for longer periods of time. Encapsulation materials suitable for cold temperatures or all extreme weather conditions should also be sought.

**Fig. 16:**
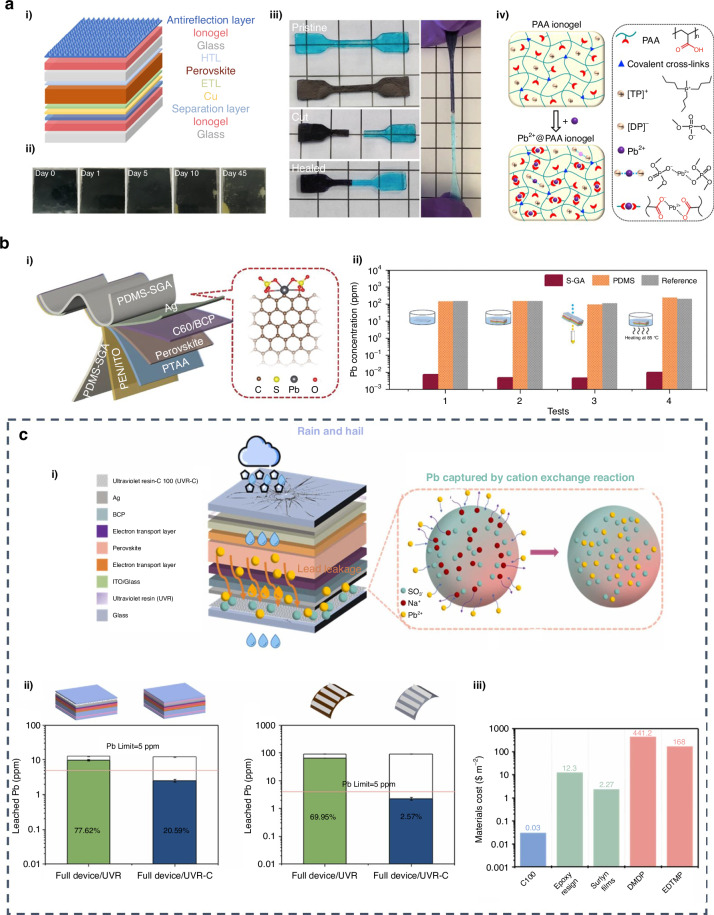
Chemical encapsulation to stop lead leakage. **a** Scheme of device structures, exploration of ionogel microstructure, and lead adsorption mechanisms, alongside progressive images illustrating the degradation of perovskite films during water soaking. Reproduced with permission from ref. ^227^ Copyright 2021 American Association for the Advancement of Science. **b** (i) Illustration depicting a flexible PSM encapsulated with a combination of S-GA and PDMS on both the front side (glass) and backside (metal) (ii) Overview of the equilibrium Pb^2+^ concentrations in various tests. Reproduced with permission from ref. ^228^ Copyright 2021 Wiley-VCH. **c** (i) Illustration depicting the encapsulation process involving cation-exchange resin, along with the molecular structure alterations before and after lead capture. (ii) Comparison of lead leaching between a rigid and flexible PSM using UVR and UVR-C as encapsulants. (iii) Evaluation of the cost associated with lead-adsorbing materials. Reproduced with permission from ref. ^229^ Copyright 2021 Elsevier Ltd

In order to carefully prevent lead from leaking out after modules have been damaged, chemical encapsulation with lead-absorbing materials is the most straightforward approach. Lead-absorbing materials usually contain phosphate^10,226,227^, carboxyl^227^, and sulfonic acid^228,229^ groups that can interact with Pb^2+^, and are coupled with encapsulation coatings with high specific surface area, such as ionogels^227^, aerogels^228^, EVA^230^, and UV resins^229^. Li et al. first reported a DMDP and EDTMP lead-trapping material containing phosphate groups and placed them on the front and back of the device, respectively (Fig. 15d)^226^. Only 0.2 ppm of lead leakage was measured in the damaged devices even when they were soaked in water for 3 h, confirming the effectiveness of chemical encapsulation. At the same time, they proposed a universal formula for calculating lead sequestration efficiency (SQE), which became a unified method for measuring the degree of lead leakage in subsequent works. Subsequently, they further attempted to prepare transparent absorbing tapes by coating DMDP on EVA film, which obtained an SQE of 99.9% in both n-i-p and p-i-n type devices (Fig. 15e)^10^. In order to meet the requirements of impact resistance and lead capture, Xiao et al. reported ionogels with lead chelating groups (phosphate and carboxyl groups) and self-healing characteristics (Fig. 16a)^227^. The encapsulated devices had only minor cracks even after being run over back and forth by a car. The damaged devices also maintained an SQE of over 99.9% after 45 days of soaking in water. All of the above studies used rigid substrates to prepare the devices, while the lead concentration obtained from flexible PSCs is more than 50 times higher than that of rigid PSCs^231^. Meanwhile, flexible devices are mostly used in portable or wearable devices, and their close contact with the human body will bring more health hazards. Therefore, for flexible devices, Li et al. used flexible sulfonated graphene aerogel combined with PDMS (PDMS-SGA) for encapsulation (Fig. 16bi)^228^. The encapsulated devices showed excellent lead capture performance, whether the damaged devices were subjected to immersion, acid rain, high-temperature tests, or the more stringent TCLP tests (Fig. 16bii). In addition, they developed a highly acidic cation exchange resin (UVR-C) for both rigid and flexible devices (Fig. 16ci)^229^. Utilizing the chelation of Pb^2+^ by the sulfonic acid group and the rapid cation exchange reaction between Pb^2+^ and Na^+^, UVR-C is able to capture Pb quickly. As shown in Fig. 16cii), it has great performance in both rigid and flexible devices. In conclusion, chemical encapsulation can hinder lead leakage on the basis of mechanical strength. However, the extra cost (Fig. 16ciii) of encapsulation cannot be ignored, and thus many researchers have turned their attention to inhibiting the lead leakage directly^229^.

#### Stopping lead leakage from devices

**Fig. 17:**
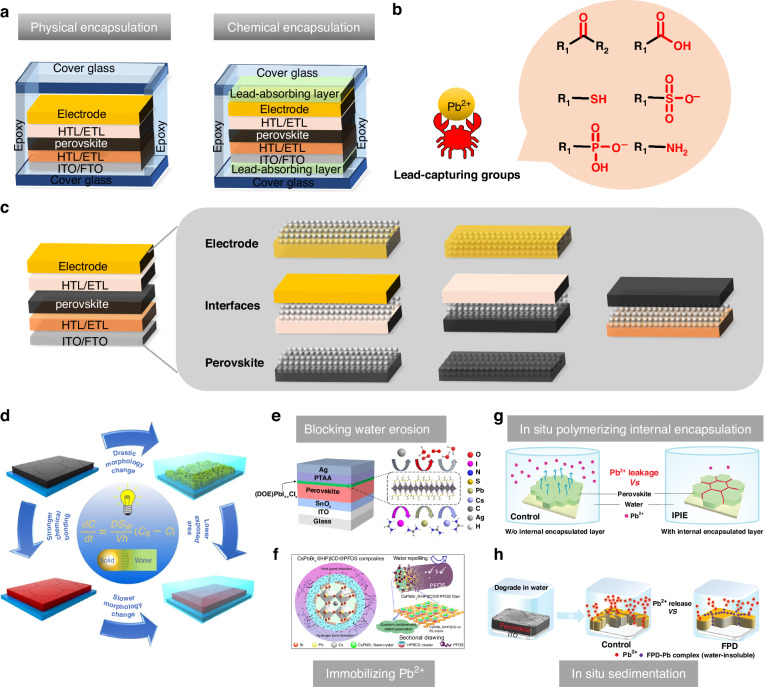
Preventing lead leakage from devices. **a** Schematic showing the physical and chemical encapsulation method. **b** Scheme of the lead-capturing groups. **c** Scheme of device structures and the functional material positions. **d** Schematic diagram of the change and modified method of perovskite film in contact with water. **e** Illustration depicting the protective capacity of (DOE)PbI_4−x_Cl_x_ in preventing both inward and outward permeation. Reproduced with permission from ref. ^245^ Copyright 2022 Wiley-VCH. **f** Schematic illustration of the chemical interaction of CsPbBr_3_@HPβCD@PFOS composites and their multifaceted functions. Reproduced with permission from ref. ^246^ Copyright 2023 Wiley-VCH. **g** Schematic diagram of the lead leakage of perovskites with and without IPIE. Reproduced with permission from ref. ^233^ Copyright 2023 Wiley-VCH. **h** Diagram illustration of the control and FPD-based perovskite degradation process in water. Reproduced with permission from ref. ^249^ Copyright 2021 Wiley-VCH

For the convenience of comparison, the various methods and effects of preventing lead leakage were organized in Table 1. Comparing the efficiency of physical encapsulation and chemical encapsulation (Fig. 17a) in preventing lead leakage, it can be found that chemical encapsulation containing lead adsorption materials plays a better effect. This indicates that materials containing lead-capturing groups play a key role. As shown in Fig. 17b, the commonly used lead capturing groups can be roughly classified into five types: carbonyl^232,233^, carboxyl^227,234^, sulfhydryl^235–238^, sulfonic acid^228,229,239–241^, and phosphoric acid groups^10,226,227,242^. They can strongly chelate Pb^2+^ and play a role in capturing or immobilizing Pb^2+^ depending on the action position. If the lead-adsorbing material is resin^239^, mesoporous scaffolds^240^, MOF^238^, sponges^243^, and other high surface area materials, the lead leakage will be less. Especially for TiO_2_ sponge, the physical deposition preparation method without chemical solvent is not only greener but also more suitable for large-area preparation. Recently, inspired by spider webs, Luo et al.^244^ implanted a multifunctional mesoporous amino-grafted carbon web between the metal electrode and cover glass to synergistically capture lead with chemical chelation and physical adsorption. With this method, they achieved Pb sequestration efficiency exceeding 99% under extreme weather conditions. As shown in Fig. 17c, the lead-segregating material can be integrated at multiple locations in the device, e.g., at the electrode and perovskite surface, inside the electrode and perovskite, at the interface of the functional layers. However, lead-segregating functionalization at or close to the perovskite surface cannot be effective in catastrophic breakdowns. This is because they do not prevent the perovskite layer from being attacked by water through horizontally formed cracks. From the chemical decomposition perspective, perovskite is transformed in contact with water into a low-dimensional hydrated perovskite, which will be further decomposed into initial components catalyzed by water, leading to lead leakage^236^.

From the perspective of dissolution behavior, due to the rapid dissolution of organic cations, the dense film transforms into a flask stacking structure (Fig. 17d), resulting in an increase in the exposed area and dissolution rate of Pb^2+^ ^245^. Therefore, internal functionalization engineering of perovskite is the key to solving the lead leakage, and the main strategies were summarized into the following four (Fig. 17e–h). The first strategy is to make perovskite water-resistant. From the perspective of modulating the dissolution behavior of Pb^2+^, Wei et al. utilized 2,2’-Dithiobis(ethylamine) cations (DOE^2+^) to convert perovskite layers into water-resistant 2D/3D perovskites in situ^245^. The dense structure of perovskite averted collapse in water, reducing the dissolution rate of Pb^2+^. In addition, the introduction of hydrophobic molecules containing F^232,236^ or long alkyl chains^246^ is also a useful method to attenuate the impact of water erosion. The second strategy is the in-situ immobilization of Pb^2+^. By introducing molecules that strongly interact with Pb^2+^ (e.g., 1,2-EDT^235^, PFDT^236^, etc.), the Pb-I bond is strengthened and the leakage of Pb^2+^ is limited. In particular, Tian et al. formed a hydrophobic shell-like in situ encapsulation by mixing CsPbBr_3_@HPβCD powder, polystyrene (PS) and perfluorooctyltriethoxysilane (PFOS)^246^. The polydentate hydroxyl groups of the cyclodextrin supramolecule strongly interact with perovskite to immobilize Pb^2+^, while the superhydrophobic fluorinated silane forms strong hydrogen bonds with the cyclodextrin molecule, further enhancing the stabilization. The leakage rate of Pb^2+^ ions from this complex structure is only ~3.94 ppt even after >3300 h of dynamic water flushing, which is much lower than the regulated Pb content in drinking water ( < 0.01 ppm) according to the World Health Organization guidelines. The third strategy is in-situ polymerization internal encapsulation (IPIE). In other words, polymers are formed at the grain boundaries and surfaces of perovskite using monomer molecules that can self-polymerize or cross-link at the perovskite annealing temperature^232,233,247,248^. The polymer network not only provides hydrophobicity similar to encapsulation but also provides multiple lead chelation sites for lead capturing or immobilization, thus effectively reducing lead leakage. The final strategy is in-situ sedimentation. PbX_2_ has a high solubility constant in water (*K*_SP_ ≈ 10^−8^). The rapid formation of low-solubility water-insoluble complexes with Pb^2+^ can directly eliminate the Pb^2+^ in water^241,249,250^. It is worth noting that the rate of formation of the water-insoluble complexes needs to be faster than the dissolution of PbX_2_. Endre Horváth et al. obtained a lead segregating efficiency of >99.9% using diammonium phosphate (DAP) with Pb^2+^ by rapid formation of insoluble phosphates^250^. Moreover, internal functionalization engineering of perovskite in conjunction with encapsulation may bring about better lead segregating efficiency^240,248^. It is important to note that any lead-segregating strategy adopted should not sacrifice device performance.

Research on lead leakage from perovskite devices has been progressing, but the corresponding test methods and standards are still imperfect. Based on the test methods summarized in Table 3, it can be seen that there is inconsistency in the area of the device tested, the method of the device is damaged and collecting dissolved Pb^2+^. Inconsistent test methods make it difficult to evaluate the extent of lead leakage. In addition, perovskite devices will be used in different scenarios and regions and then should meet different lead leakage standards. In particular, flexible devices, which are commonly used in close-to-the-body optoelectronic products, should be tested with more stringent requirements, and even require appropriate biological testing. Although the strategies discussed above can reduce lead leakage from damaged devices, they cannot prevent the irreversible decomposition of perovskite in contact with water. Fortunately, many researchers have focused on the recycling of FTO/ITO substrates^251–253^, metal electrodes^254–256^, and lead^248,257–259^ from damaged devices. Realizing the recycling of damaged devices is the key to achieving cost reduction and sustainable development. In addition, the gaps between the recycling technology and the optimal recycling potential of materials, and between laboratory recycling and industrial scale-up recycling, need to be optimized. Bridging these gaps could help to reduce energy recovery times and greenhouse gas emissions. In addition, regular module recycling provides early market entry opportunities for PSCs by addressing resource shortages and relaxing initial stability^260^.

**Table 3:** Recent progress in preventing lead leakage from Pb-based PSCs

Material	Location	Function	Damage method	Leaking method	Test method	Pb SQE	Device structure	Ref.
Control	-	-	Hail impact test (FM44787)	Water dripping tests (pH = 4.2)	ICP-MS	0	Rigid PSCs/n-i-p/Cs_0.07_FA_0.93_PbI_3_	^225^
UVR	FTO/TiO_2_/perovskite/spiro-OMeTAD/Au/glass cover with UVR at the module edges	Physical encapsulation	Hail impact test (FM44787)	Water dripping tests (pH = 4.2)	ICP-MS	6%	Rigid PSCs/n-i-p/Cs_0.07_FA_0.93_PbI_3_	^225^
ER	glass cover/ER/FTO/TiO_2_/perovskite/spiro-OMeTAD/Au/glass cover with UVR at the module edges	Physical encapsulation	Hail impact test (FM44787)	Water dripping (pH = 4.2)	ICP-MS	95%	Rigid PSCs/n-i-p/Cs_0.07_FA_0.93_PbI_3_	^225^
DMDP + EDTMP	DMDP flim/FTO/TiO_2_/perovskite/spiro-OMeTAD/Au/EDTMP/EVA	Chemical encapsulation/phosphonic acid group	Force the glass side with a hammer and use a razor blade to cut through the EVA film and the layer underneath from the back.	Water soaking	FAAS	97.70%	Rigid PSCs/n-i-p/(CsPbI_3_)_0.05_(FAPbI_3_)_0.85_(MAPbBr_3_)_0.15_	^226^
EVA + DMDP	Both sides of the as-fabricated PSCs	Chemical encapsulation/phosphonic acid group	Hail impact test (FM44787); the devices were exposed to a three-month summer outdoor condition on a rooftop	Water dripping (pH = 4.2); water soaking	FAAS + ICP-MS	99.90%	Rigid PSCs/n-i-p and p-i-n/(CsPbI_3_)_0.05_(MAPbBr_3_)_0.15_(FAPbI_3_)_0.85_	^10^
PAA@TPDP ionogel	Ionogel-1/ITO glass/PTAA/perovskite/C60/BCP/Cu/ POE/ionogel-2/glass cover	Chemical encapsulation/phosphonic acid groups and carboxyl groups	Hail impact test (FM44787); The encapsulated films were rolled over twice with a car (>1500 kg)	Water soaking	ICP-MS	>99.9%	Rigid PSCs/ p-i-n/ MA_0.7_FA_0.3_PbI_3_	^227^
PDMS-SGA	PDMS-SGA/PEN/ ITO/PTAA/perovskite/C60/BCP/Ag/PDMS-SGA	Chemical encapsulation/sulfonate groups	scratching, bending circles, crumpling, and thermal cycling	Water soaking; TCLP	ICP-OES;	99.99%; 99.73%	Flexible PSCs/p-i-n/ (FA_0.92_MA_0.08_)_0.9_Cs_0.1_Pb(I_0._92Br0.08)3	^228^
UVR-C	Glass/UVR/ITO/PTAA/perovskite/C60/BCP/Ag/UVR-C/glass	Chemical encapsulation/sulfonate groups/the rapid cation exchange reaction between Na^+^ and Pb^2+^	Hail impact test (FM44787)	Water dripping; TCLP	ICP-OES	97.3%; 79.4% (rigid PSCs)/97.4% (flexible PSCs)	Rigid and flexible PSCs/p-i-n/(FA_0.92_MA_0.08_)_0.9_Cs_0.1_Pb(I_0.92_Br_0.08_)_3_	^229^
CERs	ITO/PTAA/perovskite/C_60_/SnO_2_/carbon electrode+CER	Chemical lead-capture/sulfonate groups	Hail impact test (FM44787)	Water dripping	ICP-MS	98%	Rigid PSCs/p-i-n/Rb_0.05_Cs_0.05_FA_0.85_MA_0.05_PbI_2.85_Br_0.15_	^239^
ZrL3 MOF	ITO/PTAA/perovskite/PC61BM/ZrL3: bis-C60/ Ag	Chemical lead-capture/sulfhydryl groups	degraded PSCs	Water soaking (pH = 5.6)	ICP-OES	>80%	Rigid PSCs/p-i-n/Cs_0.05_(FA_0.85_MA_0.15_)_0.95_Pb(I_0.85_Br_0.15_)_3_	^238^
Dig+PDMS	PDMS/PEN/hc-PEDOT: PSS/PEDOT: PSS Al 4083/Perovskite/Dig/PCBM/BCP/Ag/PDMS	Chemical lead-capture/phosphonic acid groups/water-resistance	-	Water soaking	FPSMs	96%	Flexible PSCs/p-i-n/FA_0.8_MA_0.2_Pb(I_0.85_Br_015_)_3_	^242^
PAM	ITO/NiOx/PAM-perovskite/PCBM/BCP/Ag	Chemical lead-capture/the hydrogel form of polyamides	Unencapsulated module	Water soaking	FSAA	94%	Rigid PSCs/p-i-n/Cs_0.05_(FA_0.90_MA_0.10_)_0.95_Pb(I_0.90_Br_0.10_)_3_	^247^
mesoporous sulfonic acid-based resin	Glass/ITO/PTAA/resin-scaffolded perovskite/C60/BCP/Cu/resin/epoxy/plastic sheet	Chemical lead-capture and encapsulation/sulfonate groups	Hail impact test (ASTM E1038)	Water soaking	ICP-MS	95%	Rigid PSCs/p-i-n/MAPbI_3_	^240^
mesoporous amino-grafted-carbon net (BCT)	ITO/SnO2/perovskite/spiro-OMeTAD/Au/BCT/cover glass	Chemical lead-capture/physical adsorption/ carboxyl groups+ amino groups	Hail impact test (ASTM E1038)	Water soaking	ICP-OES	99%	Rigid PSCs/n-i-p/FA_0.8_MA_0.2_Pb(I_0.85_Br_0.15_)_3_	^244^
TiO_2_ sponge	ITO/PTAA/perovskite/PCBM/BCP/Au/TiO_2_ sponge	Chemical lead-capture	Unencapsulated devices	Water soaking (pH=5.8)	ICP-MS	90.20%	Rigid PSCs/p-i-n/MAPbI_3_	^243^
DPM	ITO/SnO_2_/DPM/perovskite/spiro-OMeTAD/Au	Chemical lead-capture/sulfhydryl groups	Hail impact test (FM44787)	Water dripping (pH=5.6)	lead ion testing paper	94%	Rigid PSCs/n-i-p/FA_0.92_MA_0.08_PbI_3_	^237^
L-Phe	ITO/NiOx/L-Phe/perovskite/C60/BCP/Ag	Chemical lead-capture/carboxyl groups	Films with a structure of ITO/NiO_x_ (L-Phe)	Water soaking	AES	56%	Rigid PSCs/n-i-p/FA_0.92_MA_0.08_PbI_3_	^234^
HPβCD-BTCA	Glass/ITO/HTL/HPβCD-BTCA-modified perovskite/ETL/Cu/polymer@HPβCD-BTCA-based sheet	Chemical lead-capture/water-resistance/IPIE/physical encapsulation	-	Water scouring	ICP-MS	99%	Rigid PSCs/p-i-n/Cs_0.1_FA_0.9_PbI_3_	^248^
FPD	ITO/SnO_2_/FPD-perovskite/spiro-OMeTAD/Ag.	In-situ sedimentation/porphyrin ring	-	-	ICP-MS	98%	Rigid PSCs/n-i-p/FA_0.8_MA_0.2_Pb(I_0.85_Br_0.15_)_3_	^249^
DAP	PET foil with ITO/ perovskite/ DAP mixed with zeolite and graphite on aluminum foil/PET	In-situ sedimentation	-	MAPbI_3_ was mixed with phosphate compound and then added to water	ICP-OES + ICP-MS	100%	Flexible PSCs/n-i-p/MAPbI_3_	^250^
PBSA	ITO/SnO_2_/PBSA-perovskite/spiroMeOTAD/Au	In-situ sedimentation	Unencapsulated devices	Water soaking	FAAS	86%	Rigid PSCs/n-i-p/FA_0.92_MA_0.08_PbI_3_	^241^
PFDT	FTO/c-NiO/PFDT@perovskite/PCBM/BCP/PFDT@Ag	Immobilize Pb2 + /water-resistance/sulfhydryl groups	Unencapsulated devices	Water soaking (pH = 5.5)	ICP-MS	88%	Rigid PSCs/p-i-n/Cs_0.05_(FA_0.85_MA_0.15_)_0.95_Pb(I_0.85_Br_0.15_)_3_	^236^
1,2-EDT	Glass/ITO/SnO_2_/1,2-EDT surface-treated perovskite/spiro-Ome TAD/Ag	Immobilize Pb2 + /sulfhydryl groups	Perovskite films	Water soaking	ICP-AMS	36%	Rigid PSCs/n-i-p/MAPbI_3_	^235^
POF-HDDA	ITO/SnO_2_/POF-HDDA@Perovskite/Spiro-OMeTAD/Ag	Immobilize Pb2 + /water-resistance/IPIE/carbonyl groups	Unencapsulated devices	Water soaking	ICP-OES	85%	Rigid PSCs/n-i-p/Cs_0.03_FA_0.95_MA_0.02_Pb(I_0.975_Br_0.025_)_3_	^232^
PMBAA	PET/ITO/SnO_2_/PMBAA@Perovskite/Spiro-OMeTAD/Au	Immobilize Pb^2+^/water-resistance/IPIE/carbonyl groups	Unencapsulated devices	Water soaking (pH = 5.5; 60 °C)	ICP-OES	70%	Flexible PSCs/n-i-p/Cs_0.05_FA_0.80_MA_0.15_Pb(I_0.75_Br_0.25_)_3_	^233^
2D perovskite	Glass/ITO/SnO_2_/(DOE)PbI_4-x_Cl_x_ modified-perovskite/PTAA/Ag	Water-resistance/maintain structural morphology	Unencapsulated devices	Water dripping (pH = 4.2)	ICP-MS	72%	Rigid PSCs/n-i-p/FA_0.95_Cs_0.05_PbI_3_	^245^

(Note: In-situ polymerizing internal encapsulation (IPIE)=physical encapsulation wall and chemical chelation)

### Environmental issues of toxic solvents

**Fig. 18:**
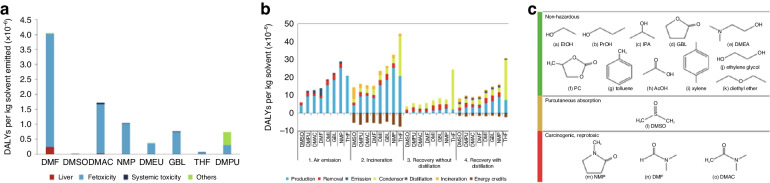
Toxic solvents. **a** Characterization of human health impacts presented as DALYs per kilogram of emitted substance in the context of urban air emissions. **b** Life cycle assessment of eight aprotic solvents for perovskite film manufacturing with four potential scenarios for EOL. Reproduced with permission from ref. ^247^ Copyright 2020 Springer Nature. **c** Diagram of rising health concerns for solvent choices in precursor dissolution. Reproduced with permission from ref. ^263^ Copyright 2016 Wiley-VCH

Solution processing technology has the advantages of simpler equipment and faster deposition speed, which is favorable for industrialized production. Therefore, most of the large-area perovskite films are prepared by it^17,261^. However, solution processing technology requires a large number of toxic solvents such as DMF and CB, which will hazard the environment and human health in the process of use, removal, emission, and end-of-life. As shown in Fig. 18, Vidal et al. investigated the adverse effects of eight commonly used polar non-protonic solvents on the human body (Fig. 18a) and the environment during the life cycle (Fig. 18b) by the USEtox method^262^. The results showed that DMF has the highest disability-adjusted life year (DALY), i.e., the highest rate of disability, followed by DMAc. At the same time, DMF has the heaviest impact during the emission process. If the whole life cycle is considered, NMP and THF have higher energy consumption in the production process and a more serious impact on the environment. DMSO has the least hazardous effect on human health and the environment, followed by DMPU. Therefore, finding non-toxic and non-polluting solvents to partially or even completely replace hazardous solvents is a challenge for the industrialization of PSCs.

#### Green solvent engineering

**Fig. 19:**
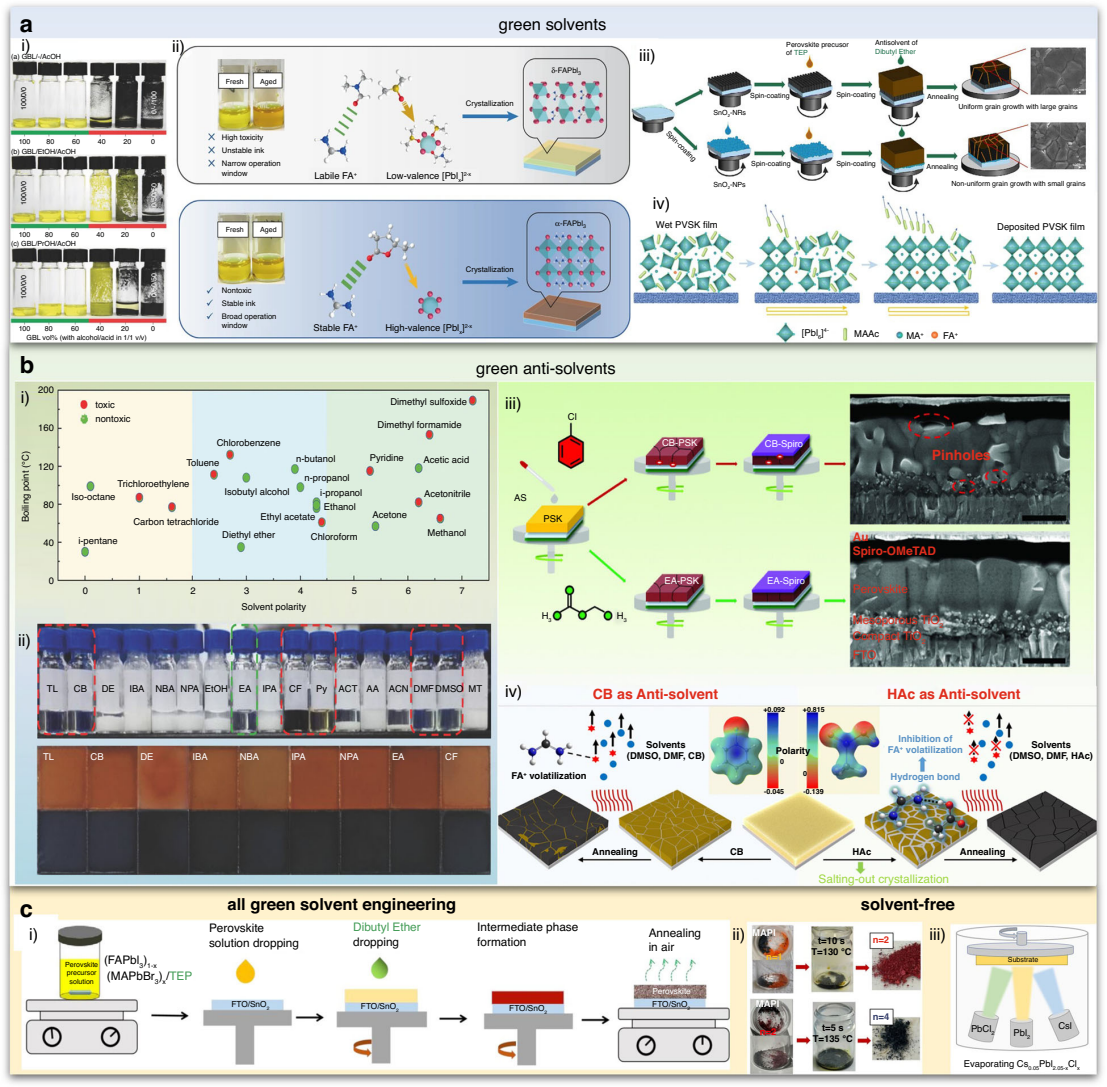
Green solvent engineering. **a** (i) Photograph of inks based on decreasing vol% of GBL with equal parts alcohol/acid. Reproduced with permission from ref. ^263^ Copyright 2016 Wiley-VCH. (ii) Comparison of the performance for DMF: DMSO and GVL-based precursor solutions. Reproduced with permission from ref. ^64^ Copyright 2023 Springer Nature. (iii) Schematic of the fabrication procedures of the perovskite with TEP and (iv) MAAc. Reproduced with permission from ref. ^161,266^ Copyright Royal Society of Chemistry and 2022 Springer Nature. **b** (i) Chart displaying the relationship between boiling point and polarity for a selection of common solvents. (ii) Photograph of various solvents dissolving the Spiro powder and extracted perovskite films. (iii) Diagram of the CB and EA solvents for the whole PSC fabrication processing. Reproduced with permission from ref. ^274^ Copyright 2013 Wiley-VCH. (iv) The comparison of preparing perovskite films with CB or HAc as an anti-solvent. Reproduced with permission from ref. ^276^ Copyright Royal Society of Chemistry. **c** (i) Schematic diagram of fabrication procedures for the all-green solvent engineering approach. Reproduced with permission from ref. ^278^ Copyright 2022 Elsevier Ltd. (ii) Liquid–solid reaction between molten layered perovskites and MAPI. Reproduced with permission from ref. ^280^ Copyright 2022 Wiley-VCH. (iii) Simplified scheme presenting the sequential vacuum deposition approach. Reproduced with permission from ref. ^281^ Copyright 2022 American Association for Advancement of Science

Figure 18c illustrates the solvents commonly used in precursor solutions which were classified according to the degree of toxicity^263^. Alcohols, esters, benzenes, GBL, and acetic acid were considered non-toxic solvents, while DMF, NMP, and DMAc were severely toxic solvents. A perfect solvent for perovskite precursors not only needs to be highly soluble in the solute, but also to form a stable intermediate phase with PbI_2_ for high-quality perovskite formation. In order to find a suitable precursor solvent, Gardner et al.^263^ attempted to mix GBL with other nontoxic solvents (Fig. 19ai) and evaluated them in terms of polarity, hydrogen bonding, and dispersion. Ultimately, the PSCs prepared with GBL/EtOH/AcOH obtained a PCE of 15.1%, which was still lower than the PCE of the DMF-prepared device (16.7%). However, GBL, although less toxic than DMF, is still regulated in some regions due to other factors. The safer and biodegradable γ-valerolactone (GVL) is structurally similar to GBL, and thus has been used as an alternative to GBL^264,265^. Recently, Miao et al.^54^ reported that eco-friendly biomass-derived green solvents containing GVL and n-butyl acetate could prepare high-quality FAPbI_3_ perovskites (Fig. 19aii). A certified efficiency of 20.23% was obtained for the micro-module (12.25 cm^2^). This is due to the stabilization of the precursor by the strong interaction between GVL and the high-valent [PbI_x_]^2-x^ complex/FA^+^. Similarly, Wu et al.^266^ obtained uniform, large-sized perovskite films by modulating the nucleation and crystallization growth process using low-toxicity triethyl phosphate (TEP) (Fig. 19aiii). In addition, the new emerging ionic liquids^267–269^ have the advantages of non-toxic, stable, easy to recycle, highly designable, and having strong interactions with Pb^2+^. Stable and viscosity-tunable perovskite inks made from methylammonium acetate (MAAc) ionic liquid solvents (Fig. 19aiv) achieved the PCE of 20.52% and 11.80% with small-area (0.05 cm^2^) and small-module (16.37 cm^2^) PSCs respectively, by screen-printing technique^161^. This provides more green solvent options for industrialization of PSCs. However, considering the utilization, a minimum of 3500 L of solvent is required for a 1-GW factory, assuming a module efficiency of 15%^270^. The low-toxicity solvents mentioned above may still need to be used with considerations for hazards such as health effects from long-term exposure and explosions. Water and ethanol are considered the most environmentally friendly solvents. Preparation of perovskites from H_2_O-based precursors (Pb (NO_3_)_2_/H_2_O^271^, PbCO_3_ NFs/H_2_O^272^) has been proven to be prospective. Ethanol-based solvent of perovskite precursors can deposit dense and homogeneous α-FAPbI_3_ films without anti-solvent dripping and thus the devices obtained a high PCE (25.1%)^273^. To develop aqueous, alcohol-based perovskite precursor solutions may be the key to solving the toxic solvent problem. In addition, green solvents such as deep eutectic solvents, cyclic carbonates converting from CO_2_, and Cyrene extracted from wood chips have been used in the chemical industry field, which may be more desirable alternatives to DMF.

#### Green anti-solvent engineering

The quality of perovskite films is directly affected by the nucleation growth process. Nonpolar solvents that do not destroy the perovskite structure are often employed as anti-solvents to remove excess precursor solvents and accelerate nucleation. However, commonly used anti-solvents (e.g., chlorobenzene, toluene, ether) are toxic and explosive due to their volatility and flammability. The search for a suitable green anti-solvent is equally urgent. Bu et al. summarized the polarity and boiling point of various solvents and indicated that the suitable polarity range of anti-solvents is 2.0-4.5 (Fig. 19bi, ii). They replaced chlorobenzene with the green solvent ethyl acetate (EA), which has a polarity of 4.4, and subsequently obtained high-quality perovskite films with no pinholes and large grains (Fig. 19biii)^274^. Recently, EA and the polymer polymethylmethacrylate were further utilized as an anti-solvent to enable NiO_x_-based inverse solar cells with *V*_OC_ close to 1.2 V, which is due to the polymer-assisted solvent promoting the growth of perovskite crystals and passivating their interfacial and intrinsic defects^275^. Besides, the green solvent acetic acid (HAc) was also applied to replace chlorobenzene. Su et al. found that HAc not only accelerated solution nucleation but also slowed down crystal growth and inhibited the loss of organic amine salts through hydrogen bonding interactions (Fig. 19biv)^276^. As a result, HAc-prepared tin-based perovskite solar cell devices brought about a PCE of 12.78%. In addition, they proposed that HAc congeners with no higher ability to form hydrogen bonds than HAc could be used as anti-solvents for the preparation of tin-based perovskite. What’s more, the green diethyl ether carbonate (DEC) has also been developed. The solvent-anti-solvent interaction modulated the p-type self-doping distribution in the tin-based perovskite, thereby optimizing the energy band structure. This resulted in a higher PCE of 14.2% than the CB-based device^277^. The above research progress confirms that chlorobenzene is not irreplaceable and more green anti-solvents can even improve the quality of perovskite.

All-green solvent engineering applying green solvents to both precursor and anti-solvent is a more desirable method. Cao et al.^278^ successfully realized the all-green solvent treatment of perovskite films by using triethyl phosphate (TEP) as the precursor solvent and combining with low-toxicity dibutyl ether (DEE) as the anti-solvent (Fig. 19ci). Due to the coordination of the phosphate group with Pb^2+^, a stable TEP-PbI_2_ intermediate phase was formed, and finally resulted in a dense and uniform film. Recently, Jangwon Seo’s research group designed an eco-friendly solvent system (GBL + methylsulfonylmethane, MSM) suitable for the scale-up preparation and immersed in green anti-solvent (BA) to produce high-quality large-area perovskite cells^279^. The prepared perovskite solar cell devices and modules can obtain a high PCE of 24% and 21.2%, respectively. This method certainly contributes to the green development of PSCs. Solvent-free preparation of perovskite is the most desirable strategy. Mercier et al.^280^ have reported that consistent melting of perovskite at moderate melting temperatures enables the preparation of layered perovskite films (Fig. 19cii). However, this method has been demonstrated so far only in monolayer perovskites (*n* = 1 of the (A)_2_(MA)_n-1_Pb_n_I_3n+1_). Meanwhile, melting perovskite requires more energy to reach a higher temperature (171 °C). Therefore, this method has yet to be improved and cost-evaluated. The most widespread solvent-free preparation technique is vapor phase deposition (Fig. 19ciii)^281^. It eliminates the need for solvents and allows precise tuning of film thickness to prepare reproducible devices. However, expensive equipment, low throughput, and high energy consumption are drawbacks of this technique, which cannot be ignored. In summary, the development of an all-green solvent system is most promising for higher environmental benefits. Additionally, the green and sustainable development of PSCs will be within reach if the solvents are further recycled periodically to close the life cycle of PSCs. Sustainable cell recycling and reuse systems will help reduce waste and resource depletion, further promoting the sustainability of PSCs.

### Recycling of PSCs

The fabrication of perovskite solar cells (PSCs) primarily involves the use of materials that are not only costly but also toxic. Neglecting to properly process these discarded devices can lead to both resource wastage and environmental contamination. An effective countermeasure is to maximize the recycling of materials from these devices, significantly mitigating environmental impact, reducing costs, and curtailing the lifecycle of the devices. Given that lead is the most harmful component in end-of-life devices, this section focuses on the recycling of lead from PSCs.

**Fig. 20:**
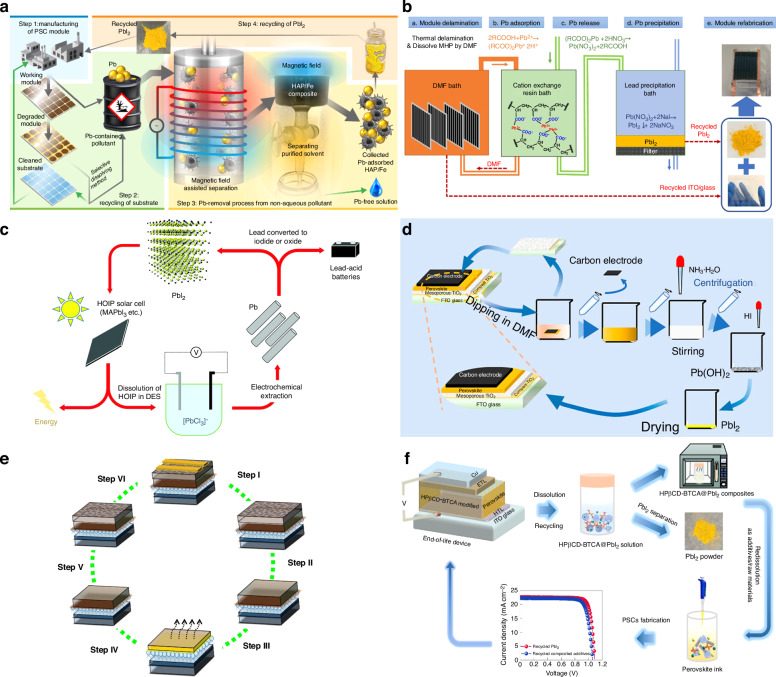
Recycling of Pb from PSC devices. **a** Illustration of the use of HAP/Fe composite for treating a Pb-containing solution pollutant and PbI_2_ regaining process after Pb removal/separation. Reproduced with permission from ref. ^259^ Copyright 2020 Springer Nature. **b** Roadmap for recycling of perovskite solar modules with CER. Reproduced with permission from ref. ^253^ Copyright 2021 Springer Nature. **c** Schematic of the deep eutectic solvent-based electrochemical recycling process, showing the route to regenerate HOIP material, or feed metallic lead back into the supply chain. Reproduced with permission from ref. ^282^ Copyright 2016 Royal Society of Chemistry. **d** Illustrated cyclic utilization process of lead from carbon-based PSCs. Reproduced with permission from ref. ^258^ Copyright 2018 American Chemical Society. **e** Schematic illustration of in situ recycling PbI_2_ from PVKSCs and the sequential fabrication of new solar cells. Reproduced with permission from ref. ^283^ Copyright 2017 Wiley-VCH. **f** Schematic illustration of Pb recycling and management in PSCs with HPβCD-BTCA. Reproduced with permission from ref. ^248^ Copyright 2023 Springer Nature

Recent studies have typically involved using polar solvents to dissolve the perovskite layer from end-of-life devices, resulting in a solution containing Pb^2+^. This is followed by the separation and extraction of PbI_2_ using adsorbents or precipitants, preparing the way for its reuse in new devices. As shown in Fig. 20a, the end-of-life devices were dissolved in DMF to obtain a Pb-containing solution, and then the Fe-decorated hydroxyapatite (HAP/Fe) hollow composite with a negative surface charge was employed to electrostatically adsorb Pb^2+^ ^259^. By leveraging solubility differences, the above-mixed material was dissolved in water, and upon the addition of KI, pure PbI_2_ can be obtained. The recycling yield through this method can reach 99.97%. Additionally, Pb^2+^ can also be adsorbed by CER, as shown in Fig. 20b^253^. Subsequently, PbI_2_ was separated and extracted by reacting with HNO_3_ solution and NaI solution with a high recycling rate of 99.99%. Enabling direct precipitation of Pb^2+^ for isolation and extraction is a simpler approach. As shown in Fig. 20c, Poll et al. used a deep eutectic solvent consisting of choline chloride and ethylene glycol to dissolve perovskite and separate Pb^2+^ directly from the solvent by electrodeposition^282^. This method allowed for the direct extraction of 99.8% Pb from the dissolved solution of perovskite. Moreover, using NH_3_·H_2_O as a chemical precipitant can achieve similar extraction results (Fig. 20d)^258^. A strategy for in situ recycling of MAPbI_3_ perovskite is shown in Fig. 20e^283^. They used tape to strip the Ag electrode, and HTL was dissolved in chlorobenzene. Then, the thermal degradation property of MAPbI_3_ was utilized to obtain a PbI_2_ layer, and the subsequent re-spin-coating of MAI solution on the PbI_2_ layer could form a new perovskite film. This strategy maintained the good performance of the devices and even the re-prepared devices (14.84%) exhibited a higher PCE than the control devices (14.35%). In order to maintain or even further improve the performance of the reprepared devices, HPβCD-BTCA which can chelate Pb^2+^ was employed as the passivator of perovskite and the Pb^2+^ adsorbent in the dissolved solution of perovskite (Fig. 20f)^248^. The HPβCD-BTCA@PbI_2_ composites can be used directly as a recycled material for reuse, and the PCE of the as-prepared devices was as high as 20%, while the PCE of the control device was only 19.63%. Additionally, PbI_2_ with a purity of 98.9% can be efficiently recovered from the HPβCD-BTCA@PbI_2_ composite material.

To conclude, the recycling of lead from end-of-life devices has seen pivotal advancements, leading to a high recycling yield and purity of PbI_2_. However, the recycling and reuse of other materials in discarded devices and the comprehensive recycling of devices have yet to be studied. In addition, the toxicity and cost of the organic solvents used in the preparation of the devices cannot be ignored. If the solvents are recycled regularly, the environmental hazards and costs of PSCs will be further reduced.

## Conclusion

In this review, we discuss the main achievements, challenges, and future prospects in the industrialization of PSCs, comprising the issues of technological limitations, multi-scenario applications, and sustainable development. Currently, PSCs have made significant strides in enhancing their efficiency, yet their industrial advancement remains hampered by critical issues—stability and upscaling. Although measures like encapsulation technology can address stability concerns related to water and oxygen, challenges persist in terms of thermal and light stability. For large-area PSMs, the crucial objective is to minimize the efficiency gap between them and laboratory-scale devices. These challenges have resulted in a notable performance gap, underscoring the pressing need for further scientific breakthroughs. As for multi-scenario applications, PSCs are available in a wide range of fabrication techniques and device structures, which can meet the application requirements of multiple scenarios. With continuous exploration and optimization, PSCs are expected to be more fully utilized in more scenarios. The sustainable development of PSCs demands our utmost attention. With useful strategies such as encapsulation, green solvents, and device recycling, the environmental impact of PSCs is effectively reduced. Nonetheless, continuous improvement remains vital to ensure that these innovative solar technologies not only serve as a cornerstone of renewable energy but also embody the essence of sustainable development.

In summary, the inherent strengths of PSCs open the door to diverse applications across various environments. While notable progress has been made in enhancing their device efficiency, conquering stability, upscaling, and sustainability issues remains pivotal for their successful integration into a wide array of scenarios. Ultimately, achieving the sustainable development of PSCs is also crucial for human society. These efforts will contribute to the widespread adoption of clean energy and the role of renewable energy in the global energy transition.
